# Fractionation of a Procyanidin-Rich Grape Seed Extract by a Preparative Integrated Ultrafiltration/Reverse Osmosis/Solid-Phase Extraction Procedure

**DOI:** 10.3390/membranes15030092

**Published:** 2025-03-14

**Authors:** Esperanza Guerrero-Hurtado, Alba Gutiérrez-Docio, Rebeca Fiedorowicz, Marin Prodanov

**Affiliations:** 1 Departamento de Producción y Caracterización de Nuevos Alimentos, Instituto de Investigación en Ciencias de la Alimentación (CIAL) (CSIC-UAM), Calle Nicolás Cabrera, 9, Campus de Cantoblanco, Universidad Autónoma de Madrid, 28049 Madrid, Spain; 2 Pharmactive Biotech Products SL, Parque Científico de Madrid, 28049 Madrid, Spain

**Keywords:** grape seed extract, procyanidin fractionation, preparative ultrafiltration, reverse osmosis, preparative solid-phase extraction

## Abstract

The consumption of grape seed extracts is known for its contribution to animal and human health and is associated with its relevant procyanidin content. However, there is a little scientific unanimity whether these properties are due to the procyanidin content or to the length of their polymers. The main reason for this doubt is the technical difficulties related to their separation. Therefore, a preparative separation of grape seed extract was carried out using an integrated ultra/diafiltration procedure with membranes of 300, 30, 5, and 1 kDa molecular mass cut-offs, reverse osmosis and solid-phase extraction to obtain fractions of very high (>300 kDa), high (300–30 kDa), intermediate (30–5 kDa), low molecular mass (5–1 kDa), very-low-mass polar molecules and ions (<1 kDa), and very-low-mass dipole molecules (<1 kDa). Process parameters, mass transfer across the membranes and the quality of separation of each fraction are described and discussed in depth. A high degree of purification was achieved for the higher-molecular-mass fractions (>300, 300–30, and 30–5 kDa), as well as the big majority of procyanidin polymers and oligomers from very-low-molecular-mass species. All fractions were characterized for their procyanidin content by normal phase high-performance liquid chromatography coupled to a photodiode array detector (NP-HPLC-PAD). This analytical technique has shown for the first time that not only do oligomeric procyanidins elute at an increasing order of elution, but polymeric ones also do the same.

## 1. Introduction

Grape seed extracts (GSEs) are obtained by the extraction of grape seeds—a key by-product of the industrial processing of grapes (*Vitis vinifera* L.) into juices and wines—using water, aqueous acetone, or aqueous ethanol mixtures. These extracts are widely recognized as nutraceutical, dietary, and feed supplements and have been firmly established in the market for several decades. They are commonly sold as either dry powders or concentrated liquids and are used as standalone products or as additives in other formulations. Both forms have seen steady growth over the years. The global market for GSE was valued at USD 175.3 billion in 2023, with a projected compound annual growth rate of 8.7% from 2024 to 2030 [1]. Several key factors are driving this growth, including the increasing recognition of GSEs as biofunctional ingredients that support cellulite reduction and weight loss, as well as the growing consumer shift toward natural-based lifestyles. In the food and beverage industry, the demand is mainly driven by the need for antimicrobial agents and plant-based antioxidants. Additionally, the surge in market demand for procyanidin-based products is closely linked to the important expansion of scientific research on GSE over the past four decades, with a record 450 publications published in 2021 [2]. A large number of these publications have shown that GSEs are primarily consumed for their health benefits in both animal [3,4] and human health [5,6]. These benefits span a wide array of bioactivities, including antioxidant, anti-inflammatory, cardioprotective, anticarcinogenic, anti-aging, antibacterial, antiulcer, and antilithiasis effects [7], as well as bone remodeling [8], among others. These bioactivities are largely attributed to the high concentrations of phenolic compounds, particularly polyphenols—also referred to as condensed tannins, polyflavan-3-ols, or procyanidins—in GSEs.

The structure of grape seed procyanidins is based on only three flavan-3-ol units, (+)-catechin (C), (+)-epicatechin (EC), and (+)-epicatechin-3-O-gallate (ECG), which are linked via C4–C8 or C4–C6 bonds [9,10]. Despite this simple foundation, grape seed procyanidins exhibit an almost limitless diversity of structures [11]. This diversity arises from several factors, including the stereochemistry and hydroxylation patterns of the flavan-3-ol units, the position of the interflavan bond, the polymer chain length (degree of polymerization), and the extent and position of esterification within the polymer chain [12]. What makes grape seed procyanidins singular is the prevalence and diversity of their galloylated forms. Species containing 1 to 8 galloyl esters have been detected [13,14], with dimers to heptamers having 1 to 5 galloyl esters [15].

Procyanidins are often divided into oligomeric and polymeric species. Waterhouse et al. [16] defined the term oligomer for polymers with a degree of polymerization of 2 to 10 for cocoa procyanidins, while Kuhnert et al. [17] defined this interval as 2 to 5 for GSE (both of them referred to ultraviolet detection at 280 nm). If detection changes to fluorescence, for cocoa procyanidins, this limit rises up to 14 elemental units [18]. So, it becomes obvious that this division of procyanidins remains somewhat arbitrary [17]. Another concern is procyanidin bioavailability. Many studies [19,20,21,22] have shown that only monomeric catechins and up to trimeric procyanidins are capable to cross human intestinal walls at trace concentrations, while bioavailability of procyanidins with a higher degree of polymerization is rather unlikely. Other studies have also claimed that the antioxidant activity of procyanidins rises along with the increase in their degree of polymerization, up to trimers, and afterwards, it decreases [23]. Searching for clinical preparations with dentin-enhancing properties from grape seeds, Bedran-Russo et al. [11] found that most active procyanidins were oligomers with a degree of polymerization from 3 to 10. All these findings suggest that low-molecular-mass oligomeric procyanidins are more interesting than those of a higher degree of polymerization. However, a main obstacle for performing biological studies in vivo is their preparative separation in sufficient amounts to provide material support for these studies.

The preparative separation of specific grape seed procyanidins is technically feasible only for oligomers with a degree of polymerization up to four. Complete separation of larger individual procyanidins becomes increasingly difficult due to the exponential rise in their structural diversity along with the increase in the polymer chain length [11]. What is achievable, however, is the separation of these compounds into fractions with different molecular masses. One of the most commonly used methods for such separations is size exclusion chromatography, based on relatively inert materials like Sephadex or Toyopearl [11,24,25,26], or preparative solid-phase extraction (SPE) chromatography [27]. A major limitation of both techniques is their relatively low resolution, yields, and reproducibility, due to accumulation of polymeric procyanidins on the head of the column [24] and even their irreversible retention in the solid phase [5]. These drawbacks require additional pre-treatments of the extracts, which further slow down the whole separation treatment. A more efficient separation method is counter-current chromatography [28], also known as centrifugal partition chromatography [29]. By employing an effective clean-up treatment involving solvent/nonsolvent precipitation and SPE, Köhler et al. [28] successfully isolated several dimeric, one trimeric, and one tetrameric singular procyanidin molecules. Similarly, Phansalkar et al. [29] used liquid/liquid extraction and centrifugal partition chromatography and obtained a purified fraction of a monogalloylated procyanidin dimer. In both cases, higher procyanidin purities were achieved, although at relatively low yields. All these examples show that the preparative separation of singular molecules from complex solutions, such as plant extracts, requires the combination of several separation techniques, which are usually based on different physicochemical phenomena, differences in molecular mass, polarity, solubility, and affinity, among others.

An alternative to the cited chromatographic techniques that can improve recovery yields is pressure-driven tangential-flow ultrafiltration/diafiltration (UF). This technique enables the efficient separation of macromolecules from smaller molecules as well as the fractionation of macromolecules based on their molecular masses. The main advantage of the use of this technique is that it allows the transformation of large volumes in a relatively short time and can be easily scalable for the treatment of bigger substrate volumes. In a previous study, we tested a 10 kDa molecular mass cut-off (MMCO) UF membrane to separate oligomeric from highly polymerized grape seed procyanidins [5]. The results demonstrated that it was possible to obtain a highly polymerized procyanidin fraction with a high degree of purification and yields of 24 g. However, this membrane allowed an important portion of the polymeric procyanidins to pass into the filtrate, resulting in a mixture with the oligomeric procyanidin. To explore the possibilities of the separation of grape seed procyanidins and to study the compositional differences between their polymers (from highly polymerized to oligomeric procyanidins), an integrated fractionation procedure was performed using four UF membranes (300, 30, 5, and 1 kDa MMCO), reverse osmosis, and solid-phase extraction. Special attention was given to key process parameters in order to optimize conditions and ensure the maximum reproducibility of the entire separation procedure.

## 2. Materials and Methods

### 2.1. Materials and Chemicals

The starting material for this study was a concentrated crude extract of sweet grape seeds (GSE) from *Vitis vinifera* L., cv. Airén, industrially produced by Output Trade S.L. (Villafranca del Penedés, Spain). The extract was obtained by the maceration of wet grape seeds in 96% ethanol and had a turbidity of 288 NTU and a total dissolved substance (TDS) content of 30%. Among the TDS, glucose, fructose and sucrose; tartaric, malic, and citric acids; and some polyols (myo-inositol) and minerals (K^+^, Ca^2+^) stand out as the main constituents. The extract was stored at 4 °C in the dark until its processing.

For the purification of the extract by UF and SPE, particle-free demineralized water was used (turbidity < 0.5 NTU and electrical conductivity of 7–8 μS/cm), obtained by a Genius 300 reverse osmosis unit (Filtec Depuradoras, Girona, Spain). The oxygen in the demineralised water was removed by displacement with N_2_ (99.99% purity, Carburos Metálicos) by bubbling it through a sintered steel diffuser for 5 min. The 96% ethanol was supplied by Panreac AppliChem (Barcelona, Spain). For the regeneration of the UF membranes, sodium hydroxide (98.8% purity) was purchased from Sigma-Aldrich (Madrid, Spain).

For the preparation of the mobile phase for HPLC analysis, acetonitrile, methanol (HPLC grade), and glacial acetic acid (AA) (99.8% purity) were used, sourced from LabKem (Barcelona, Spain), along with Milli-Q grade water, produced by a Milli-Q^®^ Integral 3 water purification system (Millipore, Bedford, MA, USA).

For the identification of catechins and dimeric procyanidins by HPLC, the following commercial reference substances were purchased: (+)-catechin (C), (+)-epicatechin (EC), (+)-epicatechin-3-gallate (ECG), procyanidin B1 [EC-(4-8)-C], and B2 [EC-(4-8)-EC] dimers (HPLC grade) from Extrasynthèse (Genay, France). Reference substances of gallic acid and ethyl gallate were supplied by Sigma-Aldrich. For the identification of non-galloylated oligomeric procyanidins (trimers to pentamers), a purified extract of cacao procyanidin oligomers (OPC) (Breko GmbH, Bremen, Germany) was used [9].

### 2.2. Experimental Equipment

The crude GSE was clarified by centrifugation in a Sorvall Lynx 6000 Superspeed centrifuge supplied by Thermo Fisher Scientific (Madrid, Spain) at 10,000× *g* and 25 °C. To further improve the clarification, the centrifuged extract was filtered through several 0.7 μm pore size MFV5 microfiber glass filters from FilterLab, Filtros Anoia SA (Barcelona, Spain), until a turbidity of <1 NTU was achieved.

The preparative fractionation of the clarified GSE into fractions of different molecular masses and polarities was carried out through integral UF and a preparative SPE procedure. For the UF, a low-pressure tangential-flow filtration system [15] was used, which was equipped with two types of membranes and corresponding membrane holders. One of these was the Prep/Scale 6 model from Millipore Merck, using three spiral-wound membranes with different MMCOs (300, 30, and 5 kDa), whose technical specifications are described in Gutiérrez-Docio et al. [5]. Subsequently, in the same filtration system, a Centramate membrane holder from Pall Co., Ltd. (Port Washington, NY, USA) was connected for the use of flat membranes packed in cassettes (4 cassettes with a total filtration area of 0.372 m^2^ (0.093 m^2^ per cassette), Omega Supor^TM^ modified polyethersulfone (PESM) membrane with a 1 kDa MMCO from Pall Co.). The diafiltration permeate from the 1 kDa MMCO membrane was subjected to concentration via reverse osmosis (RO) using a NanoMax 95 spiral-wound membrane in a high-pressure tangential-flow filtration pilot plant, as described by Morales et al. [30].

The separation of catechins and procyanidin oligomers from saccharides, di- and tricarboxylic acids, minerals, and other very polar species and ions (non-adsorbable species) was carried out in a preparative SPE unit as described by Gutiérrez-Docio et al. [5]. The resin column was prepared by passing 1 L of Amberlite™ FPX66 polymeric adsorbent (macroreticular aromatic polymer from Rohm & Haas (Philadelphia, PA, USA) suspended in 1 L of 96% ethanol) into the column by pouring the suspension from top to bottom, leaving the Teflon valve open to allow the solvent to exit freely. The resin bed packing was secured at the top with a 5 mm thick porous Nylon sponge. The purified fractions were concentrated in a pilot rotary evaporator (model RE-10V2, Labfreez Instruments Group Co., Ltd., Hunan, China) at 30 °C and a pressure of 25 mbar, and then they were dried in a LyoBeta 15 freeze-dryer from Telstar (Barcelona, Spain).

### 2.3. Analytical Equipment

**Figure 1:**
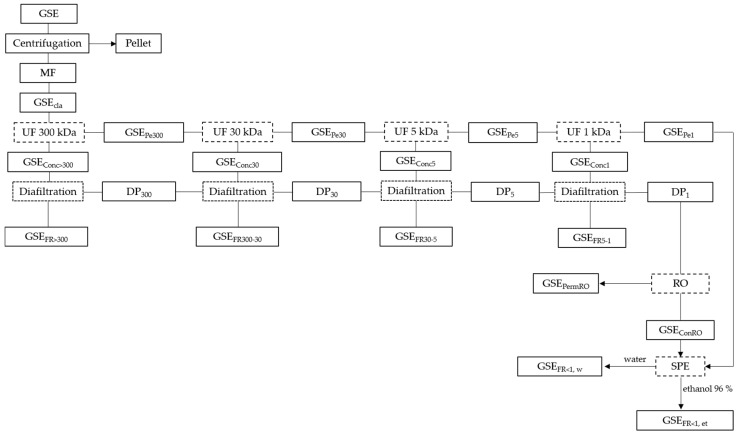
Grape seed extract (GSE) fractionation flow chart. MF—microfiltration; GSE_cla_—clarified GSE; UF—ultrafiltration; GSE_ConcX_—GSE concentrate corresponding to UF membrane X (X—300, 30, 5, 1 kDa); GSE_PeX_—GSE permeate corresponding to UF membrane X; GSE_FRX_—macromolecular fraction of GSE isolated by diafiltration with the corresponding UF membrane X; DP_X_—diafiltration permeate of GSE concentrate obtained from the corresponding membrane X; RO—reverse osmosis; GSE_PeRO_—RO permeate of DP_1_; GSE_ConcRO_—RO concentrate of DP_1_ of GSE; SPE—solid-phase extraction; GSE_FR<1,w_—very-low-molecular-mass GSE fraction obtained by SPE with water; GSE_FR<1,et_—very-low-molecular-mass fraction of GSE obtained by SPE with 96% ethanol.

For the detection and characterization of catechins, procyanidin oligomers, and polymers from the purified GSE fractions (Figure 1), liquid samples of the extract and the fractions obtained at different stages of the fractionation process, diluted in mobile phase components B (methanol/water/AA (95/3/2, *v*/*v*/*v*)) and C (water/AA, (98/2, *v*/*v*)), were prepared and analyzed using NP-HPLC (Shimadzu Corporation, Kyoto, Japan), following the methodology described by Guerrero-Hurtado et al. [31]. For the more precise identification of certain compounds, GSE and the very-low-molecular-mass fractions were analyzed by RP-HPLC according to the methodology described by Prodanov et al. [9]. In both cases, the chromatograms were acquired at 280 nm.

### 2.4. Preparative Separation Methodologies

#### 2.4.1. Clarification of Grape Seed Extract

To perform the clarification, 4.7 L of the concentrated crude GSE (30 g/100 mL of TDS) (in duplicate) was diluted with 16.5 L of demineralized water to reach a concentration of 7 g/100 mL of TDS (total volume = 21.2 L). The mixture was then subjected to clarification by centrifugation in the Sorvall Lynx 6000 centrifuge, in batches of 6 L, at 10,000× *g* for 20 min at 4 °C. The turbidity of the obtained supernatants was 8 NTU, so it was next filtered through several glass microfiber filters (0.7 μm nominal pore size). Finally, a clarified extract was obtained (0.95 NTU and 7 g/100 mL of TDS), suitable for sequential molecular separation treatment by UF, RO membranes, and SPE (Figure 1).

#### 2.4.2. Integrated UF Fractionation of Grape Seed Extract

At the beginning of each UF treatment, each membrane was conditioned by washing with demineralized water (electrical conductivity < 10 μS/cm) and subjected to hydraulic permeability tests, which were determined by comparing the variation in water flux passing through the membrane at different pressures and at a temperature of 25 °C, as described by Gutierrez-Docio et al. [5].

##### 2.4.2.1. Separation of a Very-High-Molecular-Mass Fraction (>300 kDa) by Ultra/Diafiltration of the GSE with a 300 kDa MMCO Membrane

The clarified extract (GSE_cla_) underwent an initial UF treatment using a 300 kDa MMCO membrane. Filtrations were carried out in continuous concentration mode, at pressure of 0.2 bar, with periodic additions of untreated extract into the feed tank. The resulting concentrate (GSE_Conc>300_) was subjected to diafiltration with water, where an equivalent volume of demineralized and deoxygenated water was added to the concentrate. This process was repeated as needed until the permeate was sufficiently depleted of smaller molecules and ions, indicating that the retained fraction was adequately purified. For this purification, a target endpoint of 50 μS/cm in the permeate stream was considered low enough to ensure that the very-high-molecular-mass fraction (GSE_FR>300_) was sufficiently purified. This purification threshold was applied consistently across all UF treatments in this study.

##### 2.4.2.2. Separation of a High-Molecular-Mass Fraction (300–30 kDa) by Ultra/Diafiltration of the GSE Permeate from the 300 kDa Membrane Using a 30 kDa Membrane

The permeate obtained from the 300 kDa membrane (GSE_Pe300_) was subjected to a second UF treatment using a 30 kDa MMCO membrane at a pressure of 0.5 bar. The resulting concentrate (GSE_Conc30_) underwent diafiltration with the diafiltration permeates obtained from the 300 kDa membrane treatment (DP_300_) and water, following the same methodology described in Section 2.4.2.1.

##### 2.4.2.3. Separation of an Intermediate-Molecular-Mass Fraction (30–5 kDa) by Ultra/Diafiltration of the GSE Permeate from the 30 kDa Membrane Using a 5 kDa Membrane

The permeate obtained from the 30 kDa membrane (GSE_Pe30_) was subjected to a third treatment using a 5 kDa MMCO membrane at a pressure of 0.5 bar. The resulting concentrate (GSE_Conc5_) was then diafiltered with the diafiltration permeates obtained from the 30 kDa membrane (DP_30_) and water, following the same methodology described in Section 2.4.2.1.

##### 2.4.2.4. Separation of a of Low-Molecular-Mass Fraction (5–1 kDa) by Ultra/Diafiltration of the GSE Permeate from the 5 kDa Membrane Using a 1 kDa Membrane

The permeate obtained from the 5 kDa membrane (GSE_Pe5_) underwent a fourth fractionation treatment using a 1 kDa MMCO membrane at a pressure of 1.7 bar. The resulting concentrate (GSE_Conc1_) was then diafiltered with the diafiltration permeates obtained from the 5 kDa membrane (DP_5_) and water, following the same methodology described in Section 2.4.2.1.

The concentrates obtained from each UF stage were re-concentrated by evaporation at 30 °C and 25 mbar pressure in a pilot rotary evaporator.

#### 2.4.3. Partial Concentration of a Very-Low-Molecular-Mass Fraction (<1 kDa) of the GSE by Reverse Osmosis

The 90 L of diafiltration permeates (DP_1_) obtained from the 1 kDa membrane treatment, were concentrated to 5 L by RO using the NanoMax 95 membrane at a transmembrane pressure (P_TM_) of 35.4 bar, as described by Morales et al. [30]. The concentrated very-low-molecular-mass fraction (GSE_ConRO_) was then stored frozen at −20 °C for further processing by preparative SPE.

#### 2.4.4. Membrane Regeneration

After each UF stage, the membranes were regenerated through standard washing with demineralized water, applied to both the permeate stream and the concentrate, followed by the recirculation of both permeate and concentrate streams with 0.1 N NaOH at 45 °C for 45–50 min. The used NaOH solution was then discarded, and the membrane was rinsed with demineralized water at ambient temperature until a neutral pH was achieved. Following the cleaning process, the hydraulic permeability of the membranes was evaluated, as described earlier.

#### 2.4.5. Purification of Catechins and Procyanidin Oligomers by Preparative SPE

The GSE permeates from the 1 kDa membrane (GSE_Pe1_) and the semi-concentrated diafiltration permeates by RO (GSE_ConRO_), enriched in catechins and procyanidin oligomers, were separated from saccharides (sugars and polyols), di- and tricarboxylic acids, minerals, and other highly polar and ionic species through preparative SPE in a 1 L column of Amberlite™ FPX66 adsorption resin, as described in Section 2.2. To do this, the permeate from the first replicate of the GSE fractionation by the 1 kDa membrane (GSE_Pe1_) was loaded at a flow rate of 13 mL/min. The recovered aqueous permeate was combined with the diafiltration permeate of the resin, and the joint volume (GSE_FR<1,w_) was then concentrated in a pilot rotary evaporator at 30 °C and 25 mbar pressure and stored under freezing conditions at −20 °C. The fraction enriched in dipoles (catechins and procyanidin oligomers) was desorbed with 96% ethanol (GSE_FR<1,et_), evaporated in the pilot rotary evaporator at 30 °C and 75 mbar pressure, and stored under refrigeration at 4 °C. In the same way, the permeate of the second replicate of the 1 kDa membrane and the concentration by RO (GSE_ConRO_) diafiltration permeates of the 1 kDa membrane (PD_1_) were fractionated in GSE_FR<1,w_ and GSE_FR<1,et_.

### 2.5. Statistical Analysis

The results are presented as mean values ± standard deviation (SD). Significant differences between the data were assessed using analysis of variance (ANOVA). Tukey’s least significant difference (LSD) test was used to evaluate the significance of the analysis. Differences were considered statistically significant at *p* < 0.05. All statistical tests were performed using IBM SPSS Statistics for Windows, version 25.0 (IBM Corp., Armonk, NY, USA).

## 3. Results and Discussion

### 3.1. Separation of a Very-High-Molecular-Mass Fraction (>300 kDa) by Ultra/Diafiltration of GSE_cla_ Using a 300 kDa MMCO Membrane

#### 3.1.1. Operating Conditions and Material Balance

**Figure 2:**
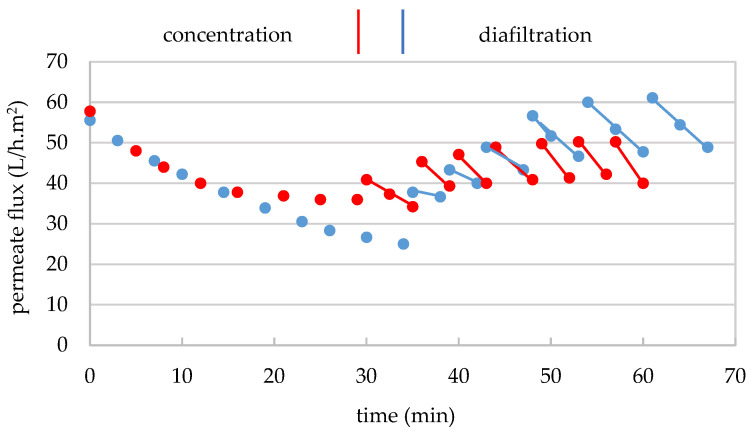
Permeate flux kinetics of the clarified GSE during ultra/diafiltration with the 300 kDa membrane: replicate 1 (blue dots) and replicate 2 (red dots) (the dots corresponding to the diafiltration range have been connected to facilitate the understanding of the results).

The separation of the very-high-molecular-mass fraction by UF of the GSE_cla_ was carried out in duplicate with a 300 kDa MMCO membrane in two stages: a macromolecule concentration stage in the feed tank and a diafiltration stage of the macromolecule-enriched concentrate with water, as described in Section 2.4.2.1. For replicate 1, an aliquot of 12 L of GSE_cla_ with 7.2 g/100 mL of TDS (Table 1) was concentrated to 1.2 L (volumetric concentration factor (F_VC_) of 10), with a permeate flux of 56 to 25 L/h.m^2^ during 34 min (Figure 2). Therefore, in the second replicate, 12 L of GSE_cla_ with 6.4 g/100 mL of TDS (Table 1) was concentrated to 1.4 L (F_VC_ of 8.6) with a permeate flux of 58 to 36 L/h.m^2^ during 28 min (Figure 2). In both cases, an important permeability loss of 55% and 37%, respectively, was observed, due to the impact of the macromolecular fraction on membrane permeability. It is important to note that the flux loss in replicate 2 was lower than in replicate 1, mainly due to the lower F_VC_ and the lower TDS content in the extract of replicate 2. After the filtration of both extracts (replicates 1 and 2), 10.8 and 10.6 L of permeates (GSE_Pe300_) were obtained, respectively (Table 1). They were stored frozen at −20 °C for subsequent treatment with the 30 kDa MMCO membrane.

Regarding the diafiltration stage, in replicate 1, 1.2 L of concentrate (GSE_Conc>300_) were subjected to six washings in another 34 min, using a total of 8 L of demineralized water (1.3 L per washing). The permeate flux ranged from 37 to 61 L/h.m^2^ (Figure 2), increasing gradually, as the content of smaller molecules (TDS) decreased during diafiltration (Figure S1), reaching higher values at the end than at the beginning of filtration. This result indicates that despite the application of such high F_VC_ (10), the successive dilutions of the concentrate allowed for the recovery of the membrane’s initial permeability. The diafiltration process was concluded when the electrical conductivity of the final permeate reached 48 μS/cm, signaling acceptable purification. A total of 7.8 L of diafiltration permeate (DP_300_) was obtained (Table 1), along with 1.2 L of concentrate from the higher-molecular-mass fraction (GSE_FR>300_). The diafiltered concentrate was freeze-dried, and the resulting dry matter was stored in the dark at 4 °C. In total, 1.83 g of dry matter from the >300 kDa fraction were obtained, which corresponds to 0.21% of the dry extract from GSE_cla_ (Table 1).

Similarly, in replicate 2 (Figure 2), 1.4 L of concentrate (GSE_Conc>300_) underwent seven washings (1.5 L of water per washing), using a total of 10.5 L of demineralized and deoxygenated water in another 32 min, with permeate flux maintained between 40 and 50 L/h.m^2^. A total of 10.5 L of diafiltration permeate (DP_300_) was collected (Table 1). In this case, the recovery of membrane permeability with the decrease in TDS in the concentrate during diafiltration was not as pronounced. However, dilution with water still helped increase the permeate flux at the start of each washing. A general slight decrease in the filtration flux was observed compared to replicate 1, most likely due to membrane fouling, which is further supported by the results from the hydraulic permeability evaluation (Figure S4). Additionally, 1.4 L of concentrate (GSE_FR>300_) were obtained, which was freeze-dried, and stored in the dark at 4 °C. After freeze-drying, 2.57 g of the >300 kDa fraction were recovered, corresponding to 0.33% of the dry extract from GSE_cla_ (Table 1).

#### 3.1.2. Mass Transfer Through the 300 kDa Membrane

##### 3.1.2.1. TDS Transfer

A very quick analysis that uses only 2 to 3 drops of extract and provides fast information on the distribution of soluble matter across the membrane is the measurement of TDS content by refractometry. The results are measured in ° Brix, which is equivalent to g/100 mL. TDS is a global measure, as it represents the sum of the specific refractive indices of all dissolved species (molecules and ions). It is also universal because all dissolved species in water respond analytically to refraction. However, there are some important drawbacks, including its low analytical sensitivity, with a quantification limit of just 0.1 g/100 mL, and its applicability only to aqueous solutions, since the refractive index of the analytes is measured relative to the refractive index of water. It is important to note that although ° Brix is widely used in the industry, it is not a measurement of the International System of Units, so along in this article the term ‘total dissolved substances (in water)’ or TDS is used.

The results from monitoring the TDS concentration in both the permeate and concentrate streams during recovery of the >300 kDa fraction show that, in the concentration stage, there was an increase in the TDS content of the concentrate from 7.2 to 8.8 g/100 mL in replicate 1 and from 6.4 to 7.4 g/100 mL in replicate 2, corresponding to a 22 and 16% enrichment, respectively (Figure S1). In both cases, it is confirmed that the 300 kDa MMCO membrane retained a relatively small amount of macromolecules. Additionally, a slight increase in TDS content was observed in the permeates, both in replicate 1 (from 6.8 to 8.2 g/100 mL) and in replicate 2 (from 5.9 to 6.4 g/100 mL), due to the simple effect of concentration in the concentrate [32]. The transfer of TDS through the membrane was generally very high, ranging from 94 to 93% in replicate 1 and from 89 to 86% in replicate 2. The latter value may indicate that the membrane did not fully recover its initial permeability.

During the diafiltration stage in both replicates, the TDS content in both, the concentrate and permeate streams decreased from 8.8 to 0.2 and from 8.2 to 0.1 g/100 mL, respectively, in replicate 1, and from 7.4 to 0.2 and from 6.4 to <0.1 g/100 mL, respectively, in replicate 2. These values suggest that the purification of macromolecules was fast, as most lower-mass molecules were removed almost entirely after the seventh washing (replicate 1) and after the sixth washing (replicate 2), with the permeate reaching 0.1 g/100 mL of TDS in both cases. However, due to the limited sensitivity of the measurements by refractometry, this was not the most suitable parameter to determine the endpoint of the diafiltration. Consequently, electrical conductivity was measured as a reference to define the completion of this stage.

##### 3.1.2.2. Electrolyte Transfer

The measurement of electrical conductivity was implemented to provide a fast assessment of electrolyte transfer across the membrane. Electrolytes primarily consist of very-low-mass ionic species, which can be considered representative of the behavior of all low-mass molecules. More importantly, conductivity measurement offers an analytical sensitivity that is 1000 times higher than that achieved by refractometry (1 µS/cm = 0.5 mg/L of TDS) [33]. This, in turn, allowed us to establish criteria for assessing the purification level of macromolecular fractions during the diafiltration process with water. Considering that distilled water has an electrical conductivity of approximately 5 µS/cm, a value of 50 µS/cm for the electrical conductivity of all permeates obtained during diafiltration in this study was established as a criterion for the purification sufficiency of each macromolecular fraction. However, since the most representative measure of mass transfer kinetics through membranes is TDS, as it reflects the contribution of all dissolved species, electrical conductivity measurements become particularly relevant during diafiltration when the TDS content falls below its detection limit of 0.1 g/100 mL, and especially when determining the endpoint of diafiltration, when the permeate conductivity reaches values below 50 µS/cm. For simplicity and to provide a clearer image, the graph only presents the electrical conductivity kinetics for replicate 1 of the GSE treatment (Figure S2).

Figure S2 shows that during the concentration stage of the extract in the first replicate, the 300 kDa membrane did not cause significant retention of electrolytes, as their transfer was complete (100–99%). The result was identical for the second replicate. During the diafiltration process, a notable decrease in electrical conductivity in the permeate stream was recorded, from 7320 to 48 μS/cm after six washings with demineralized water (replicate 1) (Table 2). This value indicates an acceptable level of purification of the macromolecular fraction from electrolytes, as well as from very-low-mass molecules. However, in replicate 2, achieving this target required seven washings to reach an electrical conductivity of 38 μS/cm (Table 2). This suggests that, in this replicate, the efficiency of the diafiltration process was affected by the decrease in the F_VC_, which led to the need for an additional washing to reach the established conductivity value.

In both cases, it is evident that fractionation by diafiltration follows a logarithmic trend, meaning that the purification effectiveness decreased as the intensity of diafiltration (number of washings) increased. Setting a target purification value can be practically useful, as achieving total purification would require an infinite number of washings.

##### 3.1.2.3. Effect on pH

During the concentration phase and the initial stages of diafiltration with the 300 kDa membrane, no notable changes in pH were observed in either the concentrate or the permeate streams in both replicates (Figure S3) (to simplify the image, the pH kinetic for only the second replicate is shown). However, from the sixth and seventh respective washings of both diafiltration replicates, a small and gradual decrease in pH was observed in the concentrates, along with a small and gradual increase in pH in the permeates. These changes were due to both the decrease in the concentrate buffer capacity caused by the replacement of electrolytes with water and the pH 6.59 of the water used in the diafiltration.

##### 3.1.2.4. Phenolic Compounds Transfer

**Figure 3:**
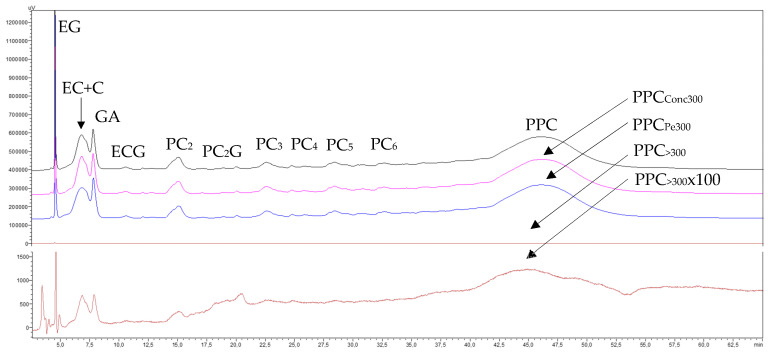
NP-HPLC chromatograms (280 nm) of the initial grape seed extract (GSE_cla_) (black), the final concentrate (>300) (pink trace), the final permeate (Pe300) (blue trace) of the concentration stage of the GSE_cla_ with the 300 kDa membrane, the final diafiltered concentrate of the very-high-molecular-mass fraction (FR > 300) (light brown flat chromatogram) and the same final diafiltered concentrate (FR > 300) magnified 100 times (brown). EG—ethyl gallate; EC–epicatechin; C—catechin; GA—gallic acid; ECG—epicatechin gallate; PC_x_—non-galloylated procyanidin oligomers; x = 2–6; PC_2_G—galloylated procyanidin dimers; PPC—procyanidin polymers; PPC_>300_—procyanidin polymers of very high molecular masses.

The NP-HPLC chromatograms shown in Figure 3 indicate that the starting GSE_cla_ was mainly composed of (in order of appearance) ethyl gallate, catechins, gallic acid, procyanidin oligomers (OPC) (from dimers to hexamers), and procyanidin polymers (PPC), which eluted below the oligomers, gradually raising the baseline until it merged into a broad peak at the end of the chromatogram. The chromatograms of the last concentrates and permeates of the concentration stage reveal that the 300 kDa membrane caused very little retention of procyanidin monomers, oligomers, and polymers, as all of them transferred from the concentrate to the permeate. However, the retention of very high-molecular-mass procyanidins (PPC_>300_) after the diafiltration stage (brown trace) was so low that the absorption response of the diafiltered concentrate (PPC_>300_) on the same scale used for the other fractions was not visible. This response had to be magnified 100 times (Figure 3, brown trace, PPC_FR>300-100_) to appreciate the separation effect. Therefore, these results confirm that the very-high-molecular-mass fraction of the GSE does contain very-high-molecular-mass procyanidins (PPC_>300_), but in a very small proportion, as shown by the mass balance data in Table 1 (0.21 and 0.33% of the GSE), as well as some impurities of catechin monomers and procyanidin oligomers of very low molecular masses.

#### 3.1.3. Evaluation of the Hydraulic Permeability of the 300 kDa Membrane After Filtration and Its Chemical Regeneration

The 300 kDa membrane exhibited a linear response in hydraulic flux within the studied pressure range (0.13–0.38 bar) (Figure S4). However, a 7% decrease in permeability was observed after regeneration following the first filtration replicate, which increased by an additional 12% after regeneration following the second filtration replicate. It is important to note that this permeability loss within just 2 h of filtration raises concerns about its long-term usability. Further studies would be needed to confirm or rule out this effect.

### 3.2. Separation of a High-Molecular-Mass Fraction (300–30 kDa) by Ultra/Diafiltration of the GSE Permeate from the 300 kDa MMCO Membrane Treatment Using a 30 kDa MMCO Membrane

#### 3.2.1. Operating Conditions and Material Balance

The separation of high-molecular-mass procyanidin polymers by UF from the GSE permeate obtained with the 300 kDa membrane treatment was performed in duplicate using a 30 kDa MMCO membrane, as described in Section 2.4.2.2. This allowed for the recovery of lower-mass molecules that passed through the 300 kDa membrane, both during the concentration and diafiltration stages. For determining the operating pressure, no permeability test was conducted on the extract in this treatment either. Therefore, the lowest possible pressure of 0.5 bar was chosen, which provided a reasonable permeate flux to complete the process within an acceptable time frame.

**Figure 4:**
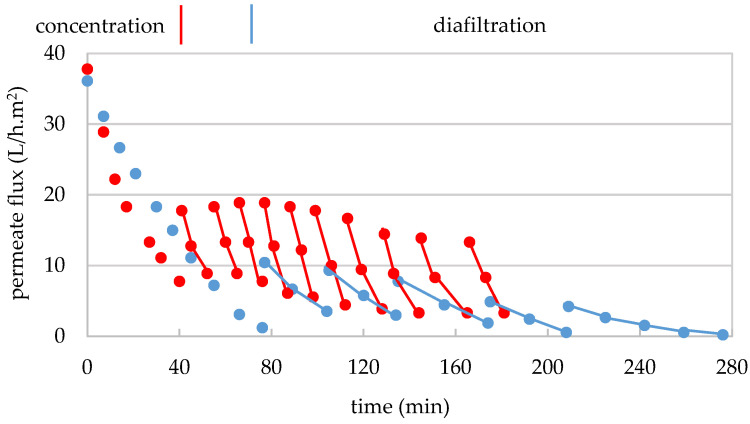
Permeate flux kinetics of the 300 kDa GSE permeate during ultra/diafiltration with the 30 kDa membrane: replicate 1 (blue dots) and replicate 2 (red dots) (the dots corresponding to the diafiltration range have been connected to facilitate the understanding of the results).

For replicate 1, 10.8 L of permeate from the first treatment with the 300 kDa membrane (GSE_Pe300_) with 7.0 g/100 mL of TDS (Table 3) were concentrated to 1.25 L (F_VC_ of 9) in 76 min. The treatment began with a permeate flux of 36.1 and ended with 1.2 L/h.m^2^ (Figure 4). Similarly, for replicate 2, 10.6 L of the second replicate of GSE_Pe300_ with 6.2 g/100 mL of TDS (Table 3) were concentrated to 1.5 L (F_VC_ of 7) in 40 min, and the permeate flux decreased from 37.8 to 7.8 L/h.m^2^ (Figure 4). In both replicates, a permeability loss of 97% and 80% was observed, respectively, which was much higher than that recorded during treatment with the 300 kDa membrane. Since the GSE input in both replicates was free from suspended solids (0.8 and 0.6 NTU, respectively), this sharp decrease in permeate flux can be primarily attributed to the impact of the macromolecules retained in the membrane or possibly to the temperature drop caused by the addition of part of the diafiltration permeate, which had not been tempered and was used cold (4–8 °C) (in replicate 1), leading to an increase in turbidity and subsequent precipitation phenomena. It is also important to note that the flux loss in replicate 2 was 17% lower than in replicate 1, mainly due to the diminishing in the F_VC_ from 9 to 7, which also resulted in a shorter treatment time of 36 min and a lower permeate flux loss compared to replicate 1. Finally, 10 and 9 L of permeates (GSE_Pe30_) were obtained from both replicates, respectively, and were stored frozen at −20 °C for subsequent treatment with the 5 kDa membrane.

For the purification of the high-molecular-mass fraction (300–30 kDa) from the remaining smaller molecules, 1.25 L of concentrate (GSE_Conc30_) from replicate 1 first underwent two washings with 3.2 L of permeate from the 300 kDa membrane diafiltration (DP_300_) (1.6 L per washing), followed by three washings with 4.8 L of demineralized and deoxygenated water (1.6 L per washing), in another 200 min. The permeate flux fluctuated between 10 and 0.2 L/h.m^2^ (Figure 4), which increased slightly during the first 2–3 washings but remained largely below 10 L/h.m^2^. This suggests that applying such a high F_VC_ (9) that leads to the retention of 73% of colloids at the end of the concentration phase can be counterproductive, as it hinders the recovery of the membrane permeability. This so-important drop in permeate flux led to the ending of the diafiltration after the fifth washing, without reaching the target final purity of 50 μS/cm in the permeate electrical conductivity. On the other hand, 6.9 L of diafiltration permeate (DP_30_) were stored at −20 °C for the next stage of ultrafiltration with the 5 kDa membrane. Additionally, 1.2 L of diafiltered concentrate (GSE_FR300–30_) were freeze-dried. In total, 71.5 g of dry matter from the 300–30 kDa macromolecular fraction were obtained, corresponding to 8.5% of the dry extract of the clarified GSE (Table 3), and stored in the dark at 4 °C. Based on the results from replicate 1, for replicate 2, a F_VC_ of 7 was used, allowing the diafiltration of 1.5 L of GSE_Conc30_ with 10 washings (1.5 L per washing), using 10.5 L of permeates (DP_Pe300_) from the 300 kDa membrane treatment in the first 7 washings and 4.5 L of demineralized water in the last 3 washings. In this case, the diafiltration was carried out with a generally higher permeate flux (18.9–3.3 L/h.m^2^) over a much shorter diafiltration time (141 min) (Figure 4). For this reason, the overall mass balance calculation for the fractionation treatment takes into account only the physicochemical data from this second replicate. Furthermore, 15 L of diafiltration permeate (DP_30_) were obtained and stored at −20 °C, and 1.5 L of concentrate (GSE_FR300–30_) were freeze-dried. In this case, 65.1 g of the high-molecular-mass fraction (300–30 kDa) were obtained, corresponding to 9.3% of the dry extract of clarified GSE (Table 3), and stored in the dark at 4 °C. In both replicates, the amount of high-molecular-mass fraction (300–30 kDa) was considerably higher than the amount of very-high-molecular-mass fraction (>300 kDa) in terms of dry matter, though it still represented a relatively small fraction.

#### 3.2.2. Mass Transfer Through the 30 kDa Membrane

##### 3.2.2.1. TDS Transfer

During the concentration stage, the TDS content in the concentrate increased from 8.2 to 29 g/100 mL in replicate 1 and from 6.6 to 14.8 g/100 mL in replicate 2 (Figure S5), corresponding to increases of 3.5 and 2.2 times, respectively. This difference is primarily due to both the higher F_VC_ (7.7 vs. 6.0) and the higher initial TDS content (7.0 vs. 6.2) applied in replicate 1. Additionally, these values indicate that, in both replicates, the 30 kDa membrane retained a higher proportion of macromolecules compared to the 300 kDa membrane (Figure S1). Furthermore, the increase in TDS content in the permeate stream, from 6.8 to 7.4 g/100 mL in replicate 1 and from 5.1 to 6.0 g/100 mL in replicate 2, was due to the concentration of TDS in the concentrate, which, as it increases, causes more TDS to pass into the permeate (concentration effect) [32]. The TDS transfer data throughout the concentration stage show a decrease from 83% to 26% in replicate 1 and from 77% to 41% in replicate 2, indicating higher retentions compared to those obtained with the 300 kDa membrane. During the diafiltration stage in both replicates (Figure S5), the TDS content in the concentrates dropped to 11.2 and 7.6 g/100 mL, and in the permeates, to 0.6 and 0.2 g/100 mL, respectively, in replicates 1 and 2. Additionally, an important decrease in TDS transfer was observed, from 61% to 5% in replicate 1 and from 51% to 3% in replicate 2, with values higher than those observed in the treatment with the 300 kDa membrane.

##### 3.2.2.2. Electrolyte Transfer

During the concentration stage, the 30 kDa membrane did not cause any significant changes in the permeate, concentrate, and electrolyte retention in either replicate. However, during the diafiltration phase, both replicates showed a gradual decrease in electrical conductivity in both concentrates and permeates. In the permeate of the first replicate, after completing five washings with 8.1 L of diafiltration permeate from the 300 kDa membrane treatment and water, the electrical conductivity reached 424 μS/cm. In the second replicate, after ten washings with a total of 15 L of diafiltration permeate from the 300 kDa membrane treatment (DP_300_) and water, the conductivity was reduced to 39 μS/cm (Table 4). In both replicates, the logarithmic trend of the electrical conductivity kinetics in the permeate flow was maintained. It is important to note that in the first replicate, the diafiltration process had to be stopped after the fifth washing due to the extremely low permeate flow. As a result, the high-molecular-mass fraction was less purified, containing a higher proportion of lower mass molecules.

##### 3.2.2.3. Effect on pH

During the concentration stage and the early stages of diafiltration, the pH kinetics in both replicates remained stable in both the concentrate and the permeate streams (Figure S6), similar to the results observed in the treatments with the 300 kDa membrane (Figure S3) (to simplify the image, the pH kinetic for only the second replicate is shown in Figure S6). However, from the sixth and fifth respective washings of both diafiltration replicates, a small and gradual decrease in pH was observed in both concentrates and a small and gradual increase in the permeates at the end of diafiltration. These changes also can be attributed to both the decrease in the buffer capacity of the concentrates caused by the replacement of the electrolytes with water and the higher pH (6.59) of the water used in diafiltration.

##### 3.2.2.4. Phenolic Compounds Transfer

**Figure 5:**
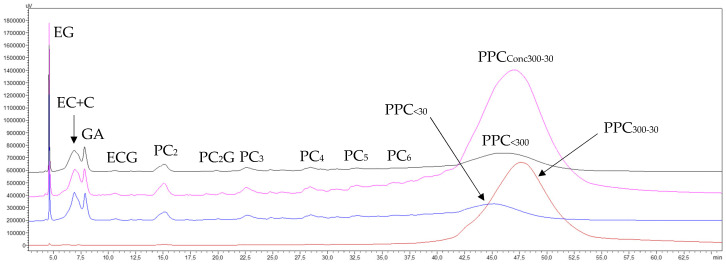
NP-HPLC chromatograms (280 nm) of the 300 kDa GSE permeate (black trace), the final concentrate (Conc300–30) (pink trace), the final permeate (Pe30) (blue trace), and the final diafiltered concentrate of the high-molecular-mass fraction (FR300–30) (brown trace). EG—ethyl gallate; EC—epicatechin; C—catechin; GA—gallic acid; ECG—epicatechin gallate; PC_x_—non-galloylated procyanidin oligomers, x = 2–6; PC_2_G—galloylated procyanidin dimers; PPC_<300—_procyanidin polymers with molecular masses below 300 kDa; PPC_Conc300–30_—concentrated high-molecular-mass procyanidin polymers with molecular masses from 300 to 30 kDa; PPC_300–30_—high-molecular-mass procyanidin polymers.

The chromatograms shown in Figure 5 reveal a high transfer rate of monomeric molecules and oligomeric procyanidins through the 30 kDa membrane, alongside a generalized concentration of all procyanidin polymers smaller than 300 kDa. Notably, there was a marked enrichment of high-molecular-mass procyanidins (PPC_<300(Conc300–30)_) (pink trace) and some loss of lower-molecular-mass procyanidin polymers (PPC_<30_) (blue trace). The chromatogram of the purified high-molecular-mass fraction (PPC_FR300–30_) (brown trace) also demonstrates that the diafiltration process eliminated most of the low-mass molecules and procyanidin oligomers, although not entirely. Furthermore, part of the lower-molecular-mass procyanidin polymers (<30 kDa) were also removed, resulting in a slight sharpening of the high-molecular-mass procyanidin polymer peak (PPC_300–30_), with minimal loss in height. This indicates a successful recovery of this polymer fraction, which is in line with the preparative recovery yields of 8.5% and 9.3% in both replicates, respectively (Table 3). In contrast, the chromatogram of the permeate (blue trace) shows that the peak corresponding to lower-molecular-mass procyanidin polymers (PPC_<30_) eluted earlier (at 45.0 min) than the peak of the purified high-molecular-mass fraction (PPC_FR300–30_) (47.7 min, brown trace). This suggests that within the peak corresponding to GSE procyanidin polymers (the permeate from the 300 kDa membrane) (46.0 min, black trace), there is an order of increasing molecular masses of its constituents, a phenomenon partially demonstrated by Spranger et al. [34].

#### 3.2.3. Evaluation of the Hydraulic Permeability of the 30 kDa Membrane After Filtration and Its Chemical Regeneration

The 30 kDa membrane demonstrated a linear response in hydraulic flux across the pressure range of 0.13–0.64 bar (Figure S7). Despite the heavy separation workload that this membrane had to endure during the first filtration, only a minor decrease in hydraulic permeability was observed after the first regeneration, which, however, was completely recovered upon membrane regeneration after completing the second filtration. These results underscore the membrane’s excellent resistance to fouling. However, further studies are needed to confirm this effect.

### 3.3. Separation of an Intermediate-Molecular-Mass Fraction (30—5 kDa) by Ultra/Diafiltration of the GSE Permeate from the 30 kDa MMCO Membrane Treatment Using a 5 kDa MMCO Membrane

#### 3.3.1. Operating Conditions and Material Balance

The operating pressure for the 5 kDa MMCO membrane was determined through a permeability test using the GSE permeate from the 30 kDa membrane, conducted within the pressure range of 0.25–0.63 bar (Figure S8). An operating pressure of 0.5 bar was selected.

As with the previous membrane treatments, the separation of procyanidin polymers with intermediate molecular masses by UF of the GSE_Pe30_ was carried out in two stages (concentration and diafiltration) using a 5 kDa MMCO membrane (in duplicate), as described in Section 2.4.2.3. This allowed for the recovery of lower-mass molecules that passed through the 300 kDa membrane, both during the concentration and diafiltration stages.

**Figure 6:**
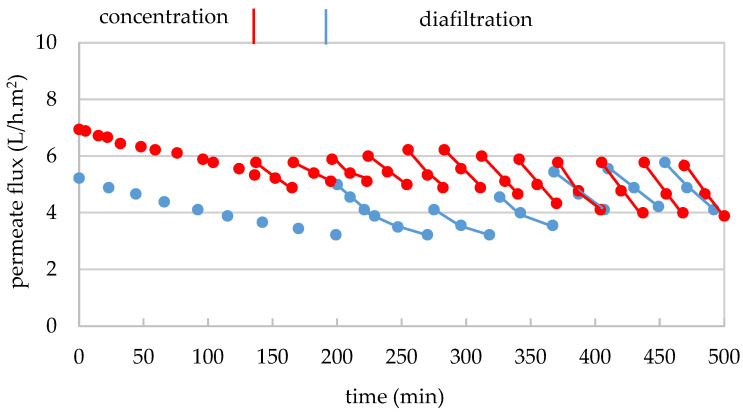
Permeate flux kinetics of the 30 kDa GSE permeate during the ultra/diafiltration with the 5 kDa membrane: replicate 1 (blue dots) and replicate 2 (red dots) (the dots corresponding to the diafiltration range have been connected to facilitate the understanding of the results).

In replicate 1, the 10 L aliquot of GSE permeate from the 30 kDa membrane (GSE_Pe30_) with 6.8 g/100 mL TDS (Table 5) were concentrated to 1.3 L (F_VC_ of 7.7) over 199 min. The treatment began with a permeate flux of 5.2 L/h.m^2^ and ended with 3.2 L/h.m^2^ (Figure 6). Similarly, in replicate 2, 9 L of GSE_Pe30_ with 6.0 g/100 mL TDS (Table 5) were concentrated to 1.5 L (F_VC_ of 6) in 136 min (Figure 6). The treatment began with a permeate flux of 6.9 L/h.m^2^ and ended with 5.2 L/h.m^2^, which corresponds to a permeability loss of 38 and 26%, respectively, which is considerably lower than the losses observed in the both previous treatments. Given that the GSE_Pe30_ was free of suspended solids in both replicates (0.21 and 0.28 NTU, respectively), the observed decrease in permeate flux can be attributed solely to the retention of macromolecules within the 30–5 kDa size range. After both filtrations, 8.7 L and 7.5 L of permeate (GSE_Pe5_) were obtained (Table 5), respectively, and stored at −20 °C for subsequent treatment with the next 1 kDa MMCO membrane.

To purify the intermediate macromolecular fraction (30–5 kDa), 1.3 L of concentrate from replicate 1 (GSE_Conc5_) were subjected to diafiltration with seven washings (1.7 L per washing). For the first four washings, 7 L of diafiltration permeate from the 30 kDa membrane (DP_30_) were used, followed by 5.2 L of demineralized and deoxygenated water in the last three washings. This process lasted for 293 min, with permeate flow maintained between 5.6 and 3.2 L/h.m^2^ (Figure 6). At the beginning of each washing, the permeate flow increased due to dilution with the diafiltration water. A total of 11.8 L of diafiltrated permeate (DP_5_) and 1.3 L of concentrate (GSE_FR30–5_) were obtained. The concentrate was freeze-dried and stored in the dark at 4 °C. In total, 135 g of dry matter from the 30—5 kDa fraction was recovered, representing 15.6% of the initial dry extract (GSE_cla_) (Table 5).

For replicate 2 (Figure 6), 1.5 L of concentrate (GSE_Conc5_) underwent 12 washings with 1.5 L per washing, using 15 L of diafiltration permeate (DP_30_) and 3 L of demineralized and deoxygenated water. This treatment took an additional 364 min, with permeate flow maintained between 6.2 and 4.7 L/h.m^2^. A total of 18 L of diafiltrated permeate (PD_5_) was collected and stored at −20 °C for subsequent treatment with the 1 kDa membrane, while 1.5 L of concentrate freeze-dried. After freeze-drying, 105 g of the 30–5 kDa procyanidin polymer fraction were recovered, representing 13.7% of the initial dry GSE (Table 5). In both replicates, the amount of dry matter from the intermediate molecular mass macromolecules was notably higher than that of the very high (>300 kDa) and high (300—30 kDa)-molecular-mass macromolecules.

#### 3.3.2. Mass Transfer Through the 5 kDa Membrane

##### 3.3.2.1. TDS Transfer

During the concentration stage (Figure S9), the TDS content in the concentrate increased from 6.8 to 12.8 g/100 mL in replicate 1 and from 6.0 to 8.4 g/100 mL in replicate 2, representing increases of 1.9 and 1.4 times, respectively. This difference is primarily due to the higher F_VC_ applied in replicate 1 (7.7 vs. 6.0 in replicate 2) and its higher initial TDS content (6.8 vs. 6.0 in replicate 2). The increase in TDS content in the permeate streams of both replicates, as seen in previous filtrations, can be attributed to the concentration effect of the concentrate. However, the TDS transfer during the concentration stage in replicate 1 was notably lower (87–53%) compared to replicate 2 (88–74%), which can be explained by the difference in both volumetric concentration factors. An appreciable increase in macromolecule retention was also observed in both replicates compared to treatments with the previous membranes. During the diafiltration stage in both replicates (Figure S9), the TDS content in the concentrates and permeates decreased to 3.3 and 0.0 g/100 mL, respectively, in replicate 1, and to 2.2 and 0.2 g/100 mL, respectively, in replicate 2.

##### 3.3.2.2. Electrolyte Transfer

During the concentration stage, the 5 kDa membrane did not cause any appreciable change in the permeate, concentrate, or electrolyte retention, as the electrolyte transfer values close to 100% were observed in both replicates. During the diafiltration phase in both replicates, a progressive decrease in electrical conductivity was observed in both the concentrates and permeates. In the permeate of the first replicate, after completing seven diafiltration washings with 12.2 L of permeates from the 30 kDa membrane treatment (DP_30_) and water, the electrical conductivity reached 109 μS/cm. In the permeate of the second replicate, after 12 washings with a total of 18 L of diafiltration permeate from the 30 kDa membrane treatment (DP) and water, an electrical conductivity of 43 μS/cm was obtained (Table 6). The logarithmic trend of the electrical conductivity measured in the permeate stream remained consistent in both replicates. It is important to note that in the first replicate, the diafiltration process had to be stopped after completing seven washings due to the important decrease in permeate flux, which resulted in recovery of a smaller purified intermediate-molecular-mass fraction, i.e., with higher impurity content. For this reason, the global material balance for the fractionation treatment was primarily based on the physicochemical data from the second replicate.

##### 3.3.2.3. Effect on pH

As observed in the previous treatments, the pH in both the concentrate and permeate streams remained stable throughout the concentration stage and up to the fourth diafiltration washing. After that, a slight increase in pH was noted in the concentrate streams, while it maintained without noticeable changes in the permeate streams (Figure S10).

##### 3.3.2.4. Phenolic Compounds Transfer

**Figure 7:**
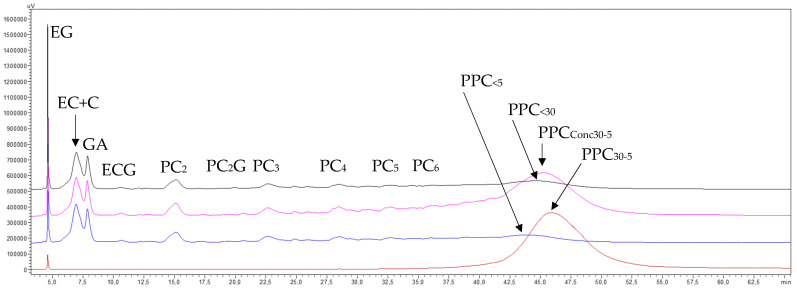
NP-HPLC chromatograms (280 nm) of the 30 kDa GSE permeate (black trace), the final concentrate (Conc30–5) (pink trace), the final permeate (Pe5) (blue trace), and the final diafiltered concentrate of the intermediate-molecular-mass fraction (FR30–5) (brown trace). EG—ethyl gallate; EC—epicatechin; C—catechin; GA—gallic acid; ECG—epicatechin gallate; PC_x_—non-galloylated procyanidin oligomers, x = 2–6; PC_2_G—galloylated procyanidin dimers; PPC_<5_—procyanidin polymers with molecular masses lower than 5 kDa; PPC_Conc30–5_—concentrated intermediate-molecular-masses procyanidin polymers with molecular masses from 30 to 5 kDa; PPC_30–5_—intermediate-molecular-mass procyanidin polymers with molecular masses from 30 to 5 kDa; PPC_<5_—procyanidin polymers with molecular masses lower than 5 kDa.

The chromatograms of the final concentrate and permeate from the UF of the GSE permeate (GSE_Pe30_) processed through the 5 kDa membrane (Figure 7) show a high transfer of monomeric molecules and procyanidin oligomers, along with a general concentration of all procyanidin polymers smaller than 30 kDa. However, there was a greater enrichment in procyanidins of intermediate molecular masses (PPC_30–5_) (pink trace) and some loss of procyanidin polymers smaller than the molecular cut-off of the membrane (PPC_<5_) (blue trace). The chromatogram of the purified intermediate-molecular-mass fraction (PPC_30–5_) (brown trace) further illustrates that the diafiltration process effectively removed the majority of lower-molecular-mass phenolic compounds and procyanidin oligomers, as well as a portion of the procyanidin polymers smaller than 5 kDa. This resulted in a noticeable enrichment of intermediate-molecular-mass procyanidins (PPC_30–5_), reflected in a higher and cleaner peak, which indicates a satisfactory recovery of this polymer fraction. These results are also consistent with the preparative recovery yields of 15.6 and 13.7% for both replicates (Table 5).

Regarding the elution order of the two newly formed macromolecular fractions, this treatment confirmed the same trend observed in the 30 kDa membrane section: the peak of procyanidin polymers smaller than 5 kDa (PPC_<5_) (blue trace) eluted first (min 43.8), followed by the peak of intermediate-molecular-mass procyanidin polymers (PPC_30–5_) (brown trace) (min 45.9). Both peaks fall within the retention time interval marked by the precursor peak of the polymers from the GSE permeate of the 30 kDa membrane (PPC_<30_, min 44.6, black trace).

#### 3.3.3. Evaluation of the Hydraulic Permeability of the 5 kDa Membrane After Filtration and Its Chemical Regeneration

The 5 kDa membrane exhibited a linear response in hydraulic permeability across the entire studied pressure range (Figure S11). Only a minor decrease of 1% was observed after the first treatment and chemical regeneration and 3% after the second treatment and chemical regeneration compared to their initial values. These results indicate that the regeneration treatment was highly effective and that the selected membrane has good resistance to fouling. However, further studies are needed to confirm continuity of this effect.

### 3.4. Separation of a Low-Molecular-Mass Fraction (5–1 kDa) by Ultra/Diafiltration of the GSE Permeate from the 5 kDa MMCO Membrane Treatment Using a 1 kDa MMCO Membrane

#### 3.4.1. Operating Conditions and Material Balance

To determine the operating pressure for the 1 kDa membrane, a permeability test was performed on the GSE_Pe5_ at pressures ranging from 0.95 to 1.7 bar (Figure S12). A pressure of 1.7 bar was chosen, as it corresponded to the highest permeate flux within the tested range and was also slightly below the manufacturer’s maximum pressure limit of 2 bar. Due to the low flux registered at this pressure, the 8.7 and 7.5 L of permeates from both replicates of the GSE_Pe5_ were divided into two sub-replicates of about 4 L aliquots, enabling fractionation of the entire GSE_Pe5_ volume in series by two sub-replicates. Each replicate within each series involved an initial concentration stage followed by a diafiltration stage, in the same manner as the previously described treatments were carried out.

**Figure 8:**
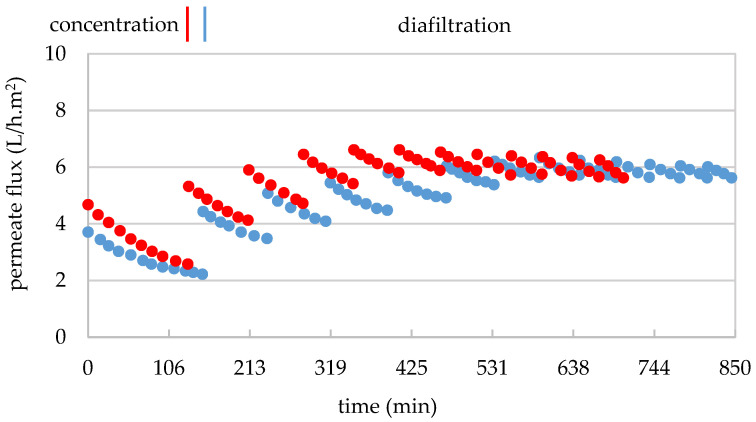
Permeate flux kinetics of the 5 kDa GSE permeate during the ultra/diafiltration with the 1 kDa membrane: sub-replicate 1′ (blue dots) and sub-replicate 2′ (red dots).

In replicate 1, a 4 L aliquot (sub-replicate 1′) of the 8.7 L permeate from the GSE_Pe5_ with 6 g/100 mL of TDS (Table 7) was concentrated to 2 L (F_VC_ of 2) over 150 min, with permeate flux rates ranging from 3.7 to 2.2 L/h.m^2^ (Figure 8). This treatment was repeated following the standard chemical regeneration of the membrane in a second treatment (sub-replicate 1″), where the remaining 4.7 L of permeate from the first replicate of the GSE_Pe5_ (with 6 g/100 mL of TDS) were concentrated to 2.3 L (F_VC_ of 2) over 180 min, with permeate flux rates ranging from 3.5 to 1.9 L/h.m^2^ (to simplify Figure 8 only the results of replicate 1 are shown). After this treatment, the membrane underwent another standard chemical regeneration. Similarly, in replicate 2, a 4 L aliquot (sub-replicate 2′) of the 7.5 L GSE_Pe5_ with 5.4 g/100 mL of TDS (Table 7) were concentrated to 2 L (F_VC_ of 2) over 131 min, with permeate flux rates ranging from 4.7 to 2.6 L/h.m^2^ (Figure 8). This treatment was repeated after another standard chemical regeneration of the membrane in a second treatment (sub-replicate 2″), where the remaining 3.5 L of permeate from the second replicate of the GSE_Pe5_ (with 5.4 g/100 mL of TDS) were concentrated to 1.8 L (F_VC_ of 2) over 83 min, with permeate flux rates ranging from 5.2 to 2.9 L/h.m^2^ (these data are very similar to the obtained for replicate 1; to simplify Figure 8 only the results of replicate 1 are shown). These results indicate a permeability loss of 41, 46, 45, and 44%, respectively, for sub-replicates 1′, 1″, 2′, and 2″, which is lower than the those observed with the 300 and 30 kDa MMCO membranes and similar to the permeability loss seen in the treatment with the 5 kDa MMCO membrane. Since the GSE_Pe5_ was free of suspended solids in four sub-replicates (0.35, 0.33, 0.44, and 0.48 NTU, respectively), the observed decrease in permeate flux can be attributed solely to the retention of macromolecules within the 5–1 kDa molecular mass range. Following the filtration process in sub-replicates 1′, 1″, 2′, and 2″, 2.0, 2.3, 2.0, and 1.5 L of permeates (GSE_Pe1_) were obtained from each sub-replicate and stored at −20 °C for subsequent SPE treatment.

In the diafiltration stage of sub-replicate 1′, 2 L of concentrate (GSE_Conc>1_) underwent 12 washings (2 L/washing), using 11.8 L of diafiltration permeate from the 5 kDa membrane treatment (DP_5_) during the first 6 washings, followed by 12.3 L of demineralised and deoxygenated water in the final 6 washings, over an additional period of 11 h and 35 min. In sub-replicate 1″, the 2.3 L of concentrate (GSE_Conc>1_) subjected to 12 washings (2.3 L/washing) with demineralized and deoxygenated water, over a further time span of 12 h and 42 min. The permeate flux gradually increased from 4.4 to 3.5 L/h.m^2^ as the washings progressed, reaching a peak of 6.3–5.7 L/h.m^2^ during the seventh washing. As observed in previous treatments, the flow rate increased as the content of lower-mass molecules (TDS) decreased during diafiltration. This resulted in higher filtration flux rates compared to those observed at the beginning of filtration, indicating that the successive dilutions of the concentrate facilitated the recovery of the membrane’s initial permeability to this substrate. Overall, the permeate flux was also enhanced at the beginning of each washing due to dilution with water. A total of 52 L of diafiltration permeate (DP_1_) were obtained from both sub-replicates 1′ and 1″, which were stored at −20 °C for subsequent RO treatment. The 2.0 and 2.3 L of the purified low-molecular-mass concentrates (GSE_FR5–1_) from sub-replicates 1′ and 1″, respectively, were freeze-dried and stored in the dark at 4 °C. In total, 83.6 g of dry matter from the 5–1 kDa fraction were obtained from sub-replicate 1′, as well as 98.7 g from sub-replicate 1″, resulting in a total of 182.3 g of dry extract from both sub-replicates 1′ and 1″ (Table 7).

In sub-replicate 2′, 2 L of GSE_Conc>1_ underwent 11 washings (2 L/washing), using 12 L of diafiltration permeate from the 5 kDa membrane treatment (DP_5_) for the first 6 washings and 10 L of demineralized, deoxygenated water for the remaining 5 washings (V_total_ = 22 L), over an additional period of 9 h and 32 min. In sub-replica 2″, 1.5 L of concentrate were subjected to 11 washings (1.5 L/washing) with demineralized, deoxygenated water, over an additional period of 7 h and 15 min. The permeate flux gradually increased from 5.3 to 4.1 L/h.m^2^ as the washings progressed, reaching a peak of 6.6–5.9 L/h.m^2^ during the fifth washing. Overall, the permeate flux was also enhanced at the start of each washing due to dilution with water. A total of 39 L of diafiltration permeate (DP_1_) were collected from sub-replicates 2′ and 2″, which were stored at −20 °C for subsequent RO treatment. The 2.0 and 1.5 L of purified low-molecular-mass concentrates (GSE_FR5–1_) obtained from sub-replicates 2′ and 2″, respectively, were freeze-dried and stored in the dark at 4 °C. In total, 80.1 g of dry matter from the 5–1 kDa fraction were obtained from sub-replicate 2′, as well as 58.1 g from sub-replicate 2″, resulting in a total of 138.2 g of dry extract from both sub-replicates 2′ and 2″ (Table 7). These results indicate an average recovery of 160.2 g, which corresponds to an average yield of 19.6% from the low-molecular-mass fraction of grape seed extract (GSE_FR5–1_).

#### 3.4.2. Mass Transfer Through the 1 kDa Membrane

##### 3.4.2.1. TDS Transfer

During the concentration stage of the GSE_Pe5_ (Figure S13), the TDS content in the concentrate stream increased from 6 to 7 g/100 mL in sub-replicate 1′ and from 5.4 to 6.3 g/100 mL in sub-replicate 2′, corresponding to an increase of 17% and 16%, respectively. The TDS content in the permeate streams remained stable, ranging from 4.2 to 4.3 g/100 mL in sub-replicate 1′ and from 3.8 to 3.9 g/100 mL in sub-replicate 2′. The TDS transfer throughout the concentration stage in both replicates showed high retention rates, reaching 31–39%, considerably higher than those observed with the tested larger MMCO membranes. The TDS kinetics for replicates 1″ and 2″ were very similar and are therefore not shown.

During the diafiltration stage of the extracts from both replicas (Figure S13), the TDS content, both in the concentrates and permeates, decreased from 7.0 to 1.5 and from 4.3 to 0.0 g/100 mL, respectively, in sub-replicate 1′, and from 6.2 to 3.9 and from 1.5 to 0.0 g/100 mL, respectively, in sub-replicate 2′. Regarding the mass transfer, a higher decrease was observed, from 57 to 5% in both replicas, considering that during each washing stage, there was a slight increase at the beginning of the treatment, followed by a decrease towards the end due to the dilution and concentration effects occurring during each washing.

##### 3.4.2.2. Electrolyte Transfer

During the concentration stage, the 1 kDa membrane did not cause appreciable changes in the electrical conductivity of the permeates, the concentrates, or in electrolyte retention, as the electrolyte transfer was approximately 90% across all four replicates. During the diafiltration phase, both the concentrates and permeates in all replicates showed a progressive decrease in electrical conductivity. In sub-replicates 1′ and 1″, after 12 diafiltration washings with 24 L of permeate from the 5 kDa membrane treatment (DP_5_) and 28 L of water, respectively, electrical conductivities of 49.2 and 50.1 μS/cm were achieved in the respective permeates (Table 8). Meanwhile, in replicate 2, after 11 diafiltration washings with a total of 22 L of permeate from the 5 kDa membrane treatment (DP_5_) and 11 washings with 17 L of water, electrical conductivities of 48.6 and 47.2 μS/cm were recorded in permeates from sub-replicates 2′ and 2″, respectively. In all replicates, the electrical conductivity measured in the permeate stream followed a logarithmic trend.

##### 3.4.2.3. Effect on pH

As observed in the previous treatments, the pH remained constant in both the concentrates and permeates across all replicates throughout the concentration stage. However, starting from the fifth diafiltration washing, a notable decrease was observed in the concentrate stream (as shown in replica 2′, Figure S13).

##### 3.4.2.4. Phenolic Compounds Transfer

**Figure 9:**
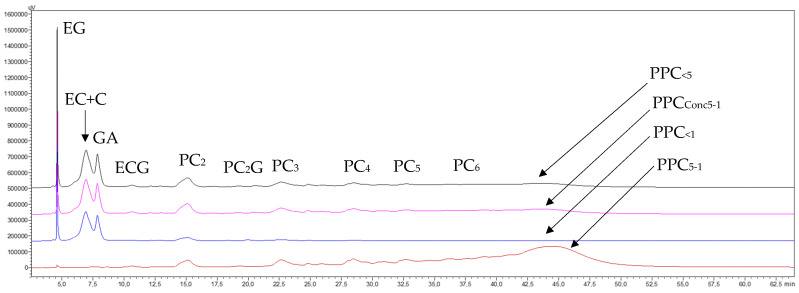
NP-HPLC chromatograms (280 nm) of the 5 kDa GSE permeate (black trace), the final concentrate (Conc5–1) (pink trace), the final permeate (Pe1) (blue trace), and the final diafiltered concentrate of the low-molecular-mass fraction (FR5–1) (brown trace) (replica 2′). EG—ethyl gallate; EC—epicatechin; C—catechin; GA—gallic acid; ECG—epicatechin gallate; PC_x_—non-galloylated procyanidin oligomers, x = 2–6; PC_2_G—galloylated procyanidin dimers; PPC_<5_—procyanidin polymers with molecular masses lower than 5 kDa; PPC_<1_—very-low-molecular-mass procyanidin polymers (less than 1 kDa); PPC_(Conc5–1)_—concentrated low-molecular-mass procyanidin polymers with masses from 5 to 1 kDa; PPC_5–1_—low-molecular-mass procyanidin polymers.

The chromatograms of the final concentrate (pink trace) and permeate (blue trace) of the ultrafiltered 5 kDa GSE permeate through the 1 kDa membrane (GSE_Pe5_) (Figure 9) show a complete transfer of only monomeric species and partial transfer of procyanidins dimers and traces of trimers, as well as a marked concentration of oligomeric procyanidins higher than dimers and low-molecular-mass procyanidin polymers (PPC_5–1_) (pink trace). The chromatogram of the purified low-molecular-mass fraction (PPC_5–1_) (brown trace) further confirms that the diafiltration process effectively removed the majority of monomeric species and a portion of the dimeric procyanidins, while enriching oligomeric procyanidins higher than dimers and low-molecular-mass procyanidin polymers (PPC_5–1_). This resulted in a much higher peak, indicating excellent recovery of this polymer fraction, which is also in line with the preparative recovery yields of 83.6% and 80.1%, respectively, for both replicas (Table 7). These results also demonstrate that the 1 kDa MMCO membrane, which is near the threshold of the nanofiltration membrane range, achieved an important retention of procyanidin oligomers. This suggests that, unlike the previous membranes, this membrane is not ideal for separating procyanidin oligomers from polymers but rather for separating both oligomers and polymers of very low molecular masses. Regarding the elution order of the two newly identified macromolecular fractions, this treatment once again confirmed the same trend observed with the 5 kDa membrane. Specifically, the peak of very-low-molecular-mass procyanidin polymers (PPC_<1_) (blue trace, Figure 9) eluted first (min 43.6), followed by the peak of low-molecular-mass procyanidin polymers (PPC_5–1_) (brown trace) at min 44.5. Both peaks fall within the retention time interval marked by the peak of their precursors, the polymers from the 5 kDa GSE permeate (min 43.6, black trace).

#### 3.4.3. Evaluation of the Hydraulic Permeability of the 1 kDa Membrane After Filtration and Its Chemical Regeneration

The 1 kDa membrane showed a linear response in hydraulic permeate flux throughout the entire studied pressure range (Figure S14). After the first treatment and chemical regeneration, permeate flux decreased gradually down to 23% compared after the last chemical regeneration. This suggests that the regeneration treatment was not very efficient. Nevertheless, the 1 kDa membrane performed exceptionally well in purifying the low-molecular-mass fraction, but it fell short in terms of productivity. Its cassette design did not allow filtration at higher pressures, leading to long operating time, making it suitable only for small-scale laboratory applications.

### 3.5. Partial Concentration of the Diafiltration Permeates of the GSE from the 1 kDa Membrane by a RO Membrane with 96% NaCl Rejection

#### 3.5.1. Operating Conditions and Material Balance

The recovery of procyanidins and other very-low-molecular-mass phenolic compounds from the diafiltration permeates generated during treatment with the 1 kDa membrane (DP_1_) was performed using a RO membrane with a 96% NaCl rejection in a single concentration run. The transmembrane pressure was set at 35.5 bar, which is very close to the maximum allowed by the manufacturer (40 bar), ensuring operation at a reasonable permeate flow rate. A total of 90 L of permeate from the four diafiltration replicates of the 1 kDa membrane treatment (DP_1_) (at 0.1 g/100 mL of TDS) were concentrated to 5.2 L (F_VC_ of 17) over a period of 259 min (Figure S16). During filtration, the permeate flux decreased from 46.5 to 12.8 L/h.m^2^, resulting in a 73% loss in permeability. This loss can be attributed to the substantial retention of minerals and very-low-molecular-mass organic molecules by the membrane during concentration. A total of 84.8 L of permeate (GSE_PermRO_) was obtained and discarded, as the main goal of this study was to recover the concentrated fraction enriched in very-low-mass molecules and ions (<1 kDa). The 5.2 L of concentrate, containing 3.8 g/100 mL of TDS, was stored at −20 °C for further purification via SPE.

#### 3.5.2. Mass Transfer Through the RO Membrane

##### 3.5.2.1. TDS Transfer

The RO concentration treatment led to an important increase in TDS content in the concentrate from 0.1 to 3.8 g/100 mL (Figure S17). This highlights the substantial retention of minerals and organic molecules by the RO membrane. TDS content remained at 0 g/100 mL in the permeate, along with a 0% TDS transfer, results that reaffirm the practical retention of the majority of molecules and ions and their concentration in the concentrate stream.

##### 3.5.2.2. Electrolyte Transfer

The RO concentration treatment led also to an important increase in the electrical conductivity of the concentrate, rising from 223 µS/cm at the start of the treatment to 9500 µS/cm at the end, indicating substantial retention of electrolytes (Figure S18). Material transfer data revealed an initial retention of 98%. However, as the concentration increased, so did the electrical conductivity in the permeate, from 5 to 606 µS/cm, which resulted in a slight increase in electrolyte transfer from 2% to 6%. This suggests that the concentration effect of the RO membrane on a solution is not constant, but rather increases with rising concentration.

Due to the limited availability of the diafiltration permeate volume generated by the 1 kDa membrane, a full follow-up to determine the maximum concentration limits of the studied RO membrane based on TDS or electrical conductivity criteria was not possible. However, despite possible variations between substrates and membranes, studies carried out by Pepper [35], Jiao et al. [36], and Rodriguez et al. [37], among others, have shown that this relationship is logarithmic, and concentrations of 25–30 g/100 mL of TDS at pressures up to 60 bar could represent reasonable maximum concentration limits. However, these data are quite contradictory to the results obtained in the present study, since reaching a DTS concentration of only 3.8 g/100 mL, going together with a loss of filtration flux of 73% and with an almost linear trend of filtration flux decrease, means that achieving concentrations of the order of those cited above may not be economically reasonable. In all cases, the obtained results suggest that RO is more favorable for treating diluted solutions in concentration operations, rather than more concentrated ones. This could prove particularly useful for the purification and recovery of diafiltration permeates.

##### 3.5.2.3. Effect on pH

Another important effect observed during the RO treatment of the GSE diafiltration permeates was the change in pH of the permeate from the outset of concentration, resulting in a 1.2-unit difference (Figure S19) with the pH of the concentrate. This effect had not been observed in any of the studied here UF membrane treatments, marking the reverse osmosis membrane as the first, to show capability to separate more acidic species from less acidic ones. Specifically, during filtration, the pH of the concentrate increased from 3.77 to 4.02, while the permeate mainly contained non-acidic species, such as simple sugars and/or polyols. It is also noteworthy that as concentration progressed, this difference began to diminish, mainly due to a decrease in the pH of the permeate from 4.90 to 4.42 by the end of the treatment.

##### 3.5.2.4. Phenolic Compounds Transfer

**Figure 10:**
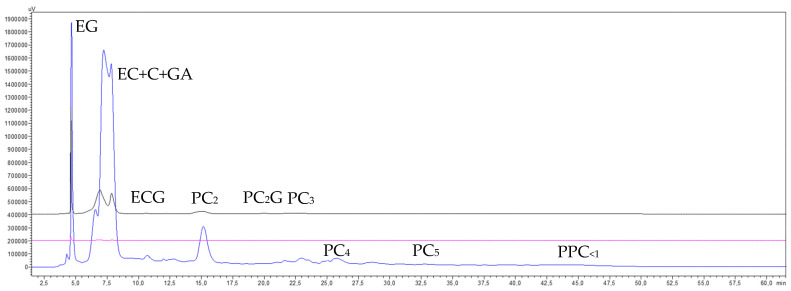
NP-HPLC chromatograms (280 nm) of the set of diafiltration permeates of GSE treated with the 1 kDa membrane (black trace) and the last permeate (GSE_Pe_) (pink trace) and last concentrate from the RO membrane. EG—ethyl gallate; EC—epicatechin; C—catechin; GA—gallic acid; ECG—epicatechin gallate; PCx—non-galloylated procyanidin oligomers, x = 2–5; PC_2_G—galloylated procyanidin dimers; PPC_<1_—procyanidin polymers of very low molecular masses.

Figure 10 shows the NP-HPLC chromatograms acquired at 280 nm for the GSE diafiltration permeates from the 1 kDa membrane, along with the final concentrate and permeate obtained after the concentration treatment using the 96% NaCl rejection RO membrane.

The chromatograms of the last concentrate (GSE_<1_) and permeate (GSE_Pe_) of the 1 kDa membrane diafiltration permeates with the 96% NaCl rejection RO membrane (Figure 10) revealed that the RO treatment produced an almost complete retention and concentration of all monomeric molecules that absorb ultraviolet light at 280 nm, as well as procyanidin oligomers up to pentamers, which had passed through the 1 kDa MMCO membrane. Therefore, it can be inferred that all, or at least most of the species that passed through the membrane were composed of very-low-mass electrolytes, since the electrical conductivity data (Figure S18) clearly indicate the transfer of electrolytes, likely in combination with monosaccharides. This is further suggested by the pH differences between the permeate and concentrate steams (Figure S19), which indicate a possible transfer of monosaccharides in the permeate. The concentration of 1 L of permeate from the RO treatment to 5 mL (200 times) in a rotary evaporator at 25 mbar of pressure and an evaporation temperature of 30 °C, followed by analysis of the concentrate by RP-HPLC, revealed the presence of some phenolic compounds, such as gallic acid, catechin, and epicatechin (Figure S20) in the permeate. These results reinforce the supposition that the primary contributors to material transfer were low-mass electrolytes, such as Na and K, or monosaccharides like glucose.

#### 3.5.3. Evaluation of the Hydraulic Permeability of 96% NaCl Rejection Reverse Osmosis Membrane After Filtration and Its Chemical Regeneration

After GSE treatment and chemical regeneration, the permeate flux increased by 16% compared to its initial values (Figure S21). This result suggests that the membrane may have been partially fouled from previous treatments or that the regeneration process was too aggressive, leading to some pore enlargement in the membrane.

### 3.6. Purification of Catechins and Procyanidin Oligomers of GSE_FR<1_ by SPE

#### 3.6.1. Operating Conditions and Material Balance

The permeates from all replicates of the two GSE series obtained through treatment with the 1 kDa membrane (GSE_Perm1_), which are equivalent to the very-low-molecular-mass fraction (GSE_FR<1_), were purified from saccharides (mainly sugars and polyols), dicarboxylic and tricarboxylic acids, minerals, and other highly polar and ionic species using preparative SPE on a 1 L Amberlite™ FPX66 adsorption resin column, according to the procedure described in Section 2.4.5. For this, 2.0 and 2.4 L of the very-low-molecular-mass fraction (GSE_FR<1_) at 4.3 and 4.4 g/100 mL TDS from the two series (1′ and 1″) of replicate 1 (Table 7 and Table 9) were consecutively loaded onto the conditioned resin at an average flow rate of 15 mL/min. A total of 4.4 L of permeate was obtained. The molecules retained by the resin were then diafiltered with approximately 2 L of demineralized and deoxygenated water at an average flow rate of 25 mL/min until 0 g/100 mL TDS (measured by refractometry) was achieved at the column outlet. A total of 2 L of diafiltration permeate were obtained, which, along with the 4.4 L of permeate, were concentrated using a pilot rotary evaporator at 30 °C and 25 mbar pressure to 520 mL at 30 g/100 mL TDS and stored frozen at −20 °C. Similarly, 2.0 and 1.8 L of the very-low-molecular-mass fraction (GSE_FR<1_) at 3.9 and 4.0 g/100 mL TDS from the two series of replicate 2 (2′ and 2″) (Table 7 and Table 9) were loaded onto the resin, resulting in 3.8 L of permeate. The molecules retained by the resin were then diafiltered with 2 L of demineralized and deoxygenated water until 0 g/100 mL TDS was reached at the column outlet. A total of 2 L of diafiltration permeate were obtained, which, along with 3.8 L of permeate, were concentrated using a pilot rotary evaporator at 30 °C and 25 mbar pressure to approximately 330 mL at 30 g/100 mL TDS and stored frozen at −20 °C. It is important to note that the fractions obtained in this way (GSE_FR<1,w_) could not be freeze-dried due to their high content of saccharides and carboxylic acids. Therefore, it was not possible to recover and measure the dry matter content, as was carried out with the UF fractions. Consequently, these fractions were concentrated to the minimum possible volume and stored at −20 °C as concentrated liquid fractions. In this case, the estimation of dry matter content was solely based on refractometry.

The fractions enriched in dipoles (phenolic compounds and procyanidin oligomers) (GSE_FR<1,et_) were desorbed with approximately 2.1 and 2.0 L of 96% ethanol, respectively, for each replicate, at an average flow rate of 15 mL/min. A total of 1.6 and 1.5 L of ethanolic fractions were obtained, respectively, for each replicate. These fractions were then subjected to distillation and concentration in a pilot rotary evaporator at 30 °C and 75 mbar pressure to recover the ethanol. The remaining aqueous concentrates were freeze-dried and stored at 4 °C. The final yield was 12.3 and 9.1 g of dry matter from the GSE_FR<1,et_ fraction. The results of the material balance after separating the fraction of dipoles from the rest of very polar and ionic species are shown in Table 9.

The same SPE procedure was applied to the 5.2 L of RO concentrate with 3.8 g/100 mL of TDS, which also corresponds to the very-low-molecular-mass fraction (GSE_FR<1_). A total of 5.2 L of permeate was collected, which, along with 2 L of diafiltrate, were concentrated using a pilot rotary evaporator at 30 °C and 25 mbar pressure to approximately 460 mL with 30 g/100 mL TDS and stored frozen at −20 °C. Additionally, 2 L of the ethanolic sub-fraction were concentrated in rotary evaporator and freeze-dried, yielding 11.1 g of dry matter from the GSE_FR<1,et_ fraction (Table 9).

**Figure 11:**
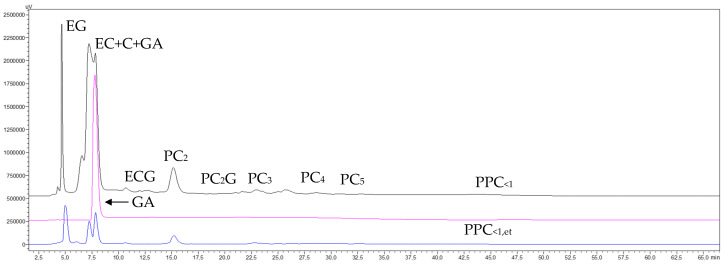
NP-HPLC (280 nm) chromatograms of the permeate from the 1 kDa membrane (GSE_Pe1_) (black trace) and the aqueous (GSE_FR<1,w_, pink trace) and ethanolic (GSE_FR<1,et_, blue trace) sub-fractions of the very-low-molecular-mass fraction, obtained by SPE by FPX66 adsorption resin. EG—ethyl gallate; EC—epicatechin; C—catechin; GA—gallic acid; ECG—epicatechin gallate; PCx—non-galloylated procyanidin oligomers, x = 2–5; PC_2_G—galloylated procyanidin dimers; PPC_<1_—of very-low-molecular-mass procyanidin polymers.

Finally, from the combined permeates of the two replicates and the diafiltration waters from the GSE treatment with the 1 kDa membrane, a total of 398 g of the aqueous sub-fraction and 32.5 g of the ethanolic sub-fraction from the very-low-molecular-mass fraction (GSE_FR<1kDa,ethanol_) were recovered by SPE. In relation to the starting grape seed extract, these two sub-fractions account for 48.4% and 3.9%, respectively (Table 9). The material balance results from this treatment also indicate a relatively low recovery of 73% for the very-low-molecular-mass fraction. This can be attributed to the retention of molecules with very low masses in the macromolecular fractions prior to treatment with the 1 kDa membrane (Figure 3, Figure 5, Figure 7 and Figure 9), the partial loss of electrolytes (particularly K, the main representative) and very-low-mass molecules (Figure 11) from the diafiltration waters during their concentration by reverse osmosis, errors in measuring dry extract content in the aqueous sub-fractions, as well as both subjective and objective challenges during the handling of the different fractions. More generally, the difficulties associated with managing volumes larger than those typically handled at the laboratory or even pilot scale, in combination with such an extensive and complex integrated fractionation process involving more than six unit operations, contributed to these discrepancies. Despite the aforementioned challenges, it is evident that SPE successfully allowed the recovery of 32.5 g of very-low-molecular-mass phenolic compounds, virtually free from saccharides and ionic species, as well as 398 g of saccharides and ionic species with some impurities of gallic acid (Figure 11).

#### 3.6.2. Phenolic Compounds Transfer

Figure 11 shows the NP-HPLC chromatograms acquired at 280 nm of the reverse osmosis concentrate of the grape seed extract permeate from the 1 kDa membrane, along with the aqueous and ethanolic sub-fractions obtained after preparative SPE of replicate 1 (only the results from replicate 1 are shown to simplify the image).

Figure 11 shows that, among all the species identified in the chromatogram of the permeate from the 1 kDa membrane (GSE_Pe1_), an important part of gallic acid passed without retention by the SPE resin and ended up in the aqueous sub-fraction of the low-molecular-mass fraction (GSE_FR<1,w_), along with other ionic species and saccharides. In contrast, the remaining phenolic compounds (monomeric molecules and procyanidin oligomers up to pentamers) were retained by the resin and later eluted with ethanol into the ethanolic sub-fraction (GSE_FR<1,et_). A further magnification of the chromatograms of the starting extract and the ethanolic sub-fraction reveals the presence of even residual procyanidin polymers with molecular masses below 1 kDa (PPC_<1_) but this image is not shown for simplicity. It is important to note that the smaller peak sizes in the chromatogram of the ethanolic sub-fraction are solely due to concentration differences, as this fraction was recovered with 2 L of ethanol, meaning it was diluted compared to the starting extract. The partial loss of gallic acid in the aqueous fraction should be regarded as inevitable because, under the conditions of direct fractionation of the GSE_Pe2_ (pH 3.77–3.94), without prior neutralization, part of the gallic acid exists in its ionic form, allowing it to pass freely through the adsorption resin. The most effective way to retain the whole of gallic acid in the ethanolic sub-fraction would be to neutralize the substrate beforehand [34] and follow the same fractionation procedure previously used. However, it is important to note that neutralization itself is a chemical intrusion that, in addition, increases the salinity of the substrate, which directly contradicts the approach of using only physical and environmentally friendly procedures.

### 3.7. Estimation of the GSE Composition

**Figure 12:**
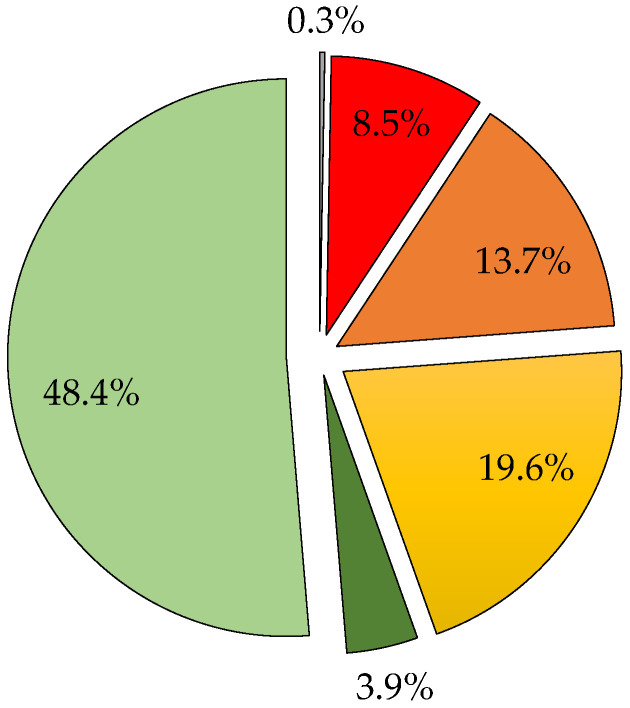
Quantitative distribution of the main constituents of GSE by molecular mass fractions (on fry matter base): very-high-mass fraction (GSE_FR>300_) (grey), high-mass fraction (GSE_FR300–30_) (red), intermediate-mass fraction (GSE_FR30–5_) (orange), low-mass fraction (GSE_FR5–1_) (yellow), very-low-mass ethanol sub-fraction (GSE_FR<1a,et_) (dark green), and very-low-mass aqueous sub-fraction (GSE_FR<1,w)_ (light green).

The material balance data for all the studied fractions (Table 1, Table 3, Table 5, Table 7 and Table 9) also allowed for a comprehensive quantitative assessment of the distribution of the main constituents of GSE according to their molecular mass, as illustrated in Figure 12.

Figure 12 shows that the predominant fraction of GSE is the aqueous sub-fraction of very low molecular masses (GSE_FR<1,w_ or GSE_FR<1,polar mol’s+ions_), which accounts for 48.4% of its total content. This fraction is characterized by a high content of saccharides (mainly sugars and polyols), di- and tricarboxylic acids (data not shown), and other unidentified very polar and ionic species (minerals). It can also be seen that the set of monomeric phenolic compounds and procyanidin dimers and trimers constitutes only 3.9% (GSE_FR<1,et_ or GSE_FR<1,dipoles_) of the total GSE content. Among the phenolic compounds, procyanidin oligomers larger than trimers and polymers with higher molecular masses (GSE_FR300–30_, GSE_FR30–5,_ GSE_FR5–1_) are the most abundant (41.8%). Within the procyanidin polymers, predominant was the low-molecular-mass fraction (GSE_FR5–1_) with 19.6%, followed by the intermediate-molecular-mass fraction (GSE_FR30–5_), the high-molecular-mass fractions (GSE_FR300–30_), and the very-low-molecular-mass ethanolic sub-fraction (GSE_FR<1,dipoles_). The very-high-molecular-mass fraction (GSE_FR>300_) stands out for its minimal proportion (0.3%). Despite the important variations in the proportions of procyanidins across the fractions of different molecular masses, as shown in Figure 12, and the inherent approximation due to the difficulty in subtracting potential polysaccharides and proteins, it can be concluded that procyanidins are distributed across all the studied fractions, including those with very high and very low molecular masses.

## 4. Conclusions

The results obtained in this study suggest that ultra/diafiltration with membranes of MMCO > 5 kDa is the ideal preparative separation technique for purifying and isolating macromolecular fractions from smaller molecules and ions. The used procedure allowed for the recovery of fractions in quantities ranging from 4 to 450 g with reasonable time and purification efficiency. However, the complete removal of smaller molecules and ions from the corresponding macromolecules through diafiltration follows a decreasing exponential trend, indicating that the transfer of small molecules across the membranes diminishes as their concentration in the concentrates decreases. This implies that, despite using exhaustive diafiltration intensities, achieving complete separation of these fractions is practically impossible. Therefore, the ultra/diafiltration technique can provide reasonably satisfactory purification results, as long as very high purity criteria are not the primary objective. Achieving such high purity would require longer diafiltration times, which would increase costs and generate larger volumes of diafiltration effluents. Furthermore, the proposal of objectives for achieving the greater purification of macromolecules by diafiltration contradicts the aim of obtaining more homogeneous macromolecular fractions in terms of molecular masses. This is because more intensive diafiltration leads to the transfer of larger molecules into lower-molecular-mass fractions. Therefore, it is crucial to define an optimal balance between achieving sufficient purification and obtaining more uniform macromolecular fractionations. To establish the limits of this balance, electrical conductivity measurement has been adopted as a quick, effective, and reliable analytical tool, which can even be implemented on-line during the diafiltration process itself. The obtained results indicate that, for achieving an adequate purification of macromolecular fractions through diafiltration, electrical conductivities from 30 to 200 µS/cm in the filtrate stream need to be achieved, depending on the MMCO of the used membrane. These values are attained through more intensive diafiltration, with lower-MMCO membranes requiring more extensive processing. For the same reasons, higher fraction purities can be obtained when purifying higher-molecular-mass procyanidins compared to lower-molecular-mass procyanidins.

Conversely, the use of ultrafiltration membranes with very low MMCOs, approaching the nanofiltration threshold, such as the used 1 kDa membrane, results in the relevant retention of procyanidin oligomers in the macromolecular fraction. This suggests that membranes of similar MMCOs, unlike the more open ones, are not suitable for separating procyanidin polymers from oligomers. Instead, they separate a mixture of procyanidin polymers and oligomers from very-low-molecular-mass species, including catechins and procyanidin dimers. In these cases, some loss of procyanidin oligomers in the permeate must be assumed, or membranes with smaller pore sizes from the nanofiltration range should be explored.

Another key technological factor that helps prevent precipitation and maintain an acceptable filtration flux is defining a volumetric concentration factor (F_VC_) that aligns with the physicochemical properties of both the grape seed extract and the used membranes. For membranes with MMCOs of 300 and 30 kDa, an F_VC_ < 7 should be applied; for membranes with an MMCO around 5 kDa, an F_VC_ < 6 should be applied; and for membranes with an MMCO around 1 kDa, an F_VC_ < 2 should be applied.

Reverse osmosis treatment has proven highly effective for recovering of molecules from diafiltration permeates, which are characterized by a low total dissolved substance (TDS) content. This, in turn, has made this treatment both faster and more efficient.

The recovery of ultrafiltration and reverse osmosis membrane permeability using a standard regeneration procedure, consisting of multiple washings with water at 45 °C, followed by chemical regeneration with a 0.1 N NaOH solution and subsequent rinsing to achieve a neutral pH, led to the satisfactory recovery of 30 kDa and 5 kDa membranes. However, for the other studied membranes, irreversible fouling was observed after filtration and regeneration. Further studies are needed to expand the current knowledge on the recovery of membrane permeability after use.

The sequential use of ultrafiltration membranes and solid-phase extraction has been crucial in separating dipoles (procyanidins and other phenolic compounds) from very polar molecules and ions (sugars, dicarboxylic and tricarboxylic acids, minerals) present in grape seed extracts.

To evaluate the degree of purification of procyanidin oligomers and polymers in grape seed extracts, NP-HPLC-PAD has proven to be the most suitable analytical technique. Moreover, the combined use of preparative fractionation of procyanidin polymers via ultra/diafiltration, solid-phase extraction, and NP-HPLC-PAD has, for the first time, demonstrated that, within the broad peak corresponding to chromatographically non-separable procyanidin polymers eluting at the end of the NP-HPLC chromatogram, the same elution order of increasing molecular masses is followed as with procyanidin oligomers. However, for the individual analysis of very-low-molecular-mass phenolic compounds, the most appropriate technique is RP-HPLC. On the other hand, the sequential use of preparative integrated ultra/diafiltration, reverse osmosis, and solid-phase extraction has enabled the evaluation of the overall content of each of the recovered fraction and, from there, defining the proportion that these groups of compounds occupy in the dry matter of industrial grape seed extract.

## Figures and Tables

**Table 1:** Material balance of the fractionation of the GSE_cla_ by the 300 kDa membrane in both replicates.

GSE_cla_	V_GSE_	TDS_GSEcla_	ms,_GSEcla_	ms,_FR>300_	Pro GSE_cla_	V_Pe300_	TDS_Pe300_	ms_,Pe300_
	(L)	(g/100 mL)	(g)	(g)	(%)	(L)	(g/100 mL)	(g)
Rep 1	12	7.2	864	1.83	0.21	10.8	7.0	756
Rep 2	12	6.4	768	2.57	0.33	10.6	6.2	657

V—volume; TDS—total dissolved substances; ms—dry matter; FR—fraction; Prop—proportion of GSE; GSE_cla_—clarified grape seed extract; Pe300—300 kDa permeate; Rep—replicate.

**Table 2:** Diafiltration intensity and electrical conductivity achieved in the permeates of both replicates, at the end of the diafiltration of the very-high-molecular-mass fraction (GSE_FR>300_) of the clarified grape seed extract by the 300 kDa membrane.

Diafiltration GSE_FR>300kDa_	Diafiltration Intensity (Number of Washings)	Electrical Conductivity (μS/cm)
Replicate 1	6	48
Replicate 2	7	38

**Table 3:** Material balance of the fractionation of the GSE_Pe300_ by the 30 kDa membrane in both replicates.

Pe_300_	V_Pe300_	TDS_Pe300_	ms_,Pe300_	ms_,FR300–30_	Pro GSE_cla_	V_Pe30_	TDS_Pe30_	ms_,Pe30_
	(L)	(g/100 mL)	(g)	(g)	(%)	(L)	(g/100 mL)	(g)
Rep 1	10.8	7.0	756	71.5	8.5	10.0	6.8	680
Rep 2	10.6	6.2	657	65.1	9.3	9.0	6.0	540

V—volume; TDS—total dissolved substances; ms—dry matter; FR—fraction; Pro GSE_clar_—proportion of the clarified GSE; Pe300—300 kDa permeate; Rep—replicate.

**Table 4:** Diafiltration intensity and electrical conductivity achieved in the permeates of both replicates at the end of the diafiltration of the high-molecular-mass fraction (GSE_FR300–30_) of the 300 kDa GSE permeate with the 30 kDa MMCO membrane.

Diafiltration GSE_FR300–30_	Diafiltration Intensity (Number of Washings)	Electrical Conductivity (μS/cm)
Replicate 1	5	424
Replicate 2	10	39

**Table 5:** Material balance of the fractionation of the GSE_Pe30_ by the 5 kDa membrane in both replicates.

Pe30	V_Pem30_	TDS_Pe30_	ms,_Pe30_	ms_,FR30–5_	Pro GSE_cla_	V_Pe5_	TDS_Pe5_	ms_,Pe5_
	(L)	(g/100 mL)	(g)	(g)	(%)	(L)	(g/100 mL)	(g)
Rep 1	10.0	6.8	680	135	15.6	8.7	6.0	522
Rep 2	9.0	6.0	540	105	13.7	7.5	5.4	405

V—volume; TDS—total dissolved substances; ms—dry matter; FR—fraction; Pro GSE_clar_—proportion of the clarified GSE; Pe30—30 kDa permeate; Rep—replicate.

**Table 6:** Diafiltration intensity and electrical conductivity achieved in the permeates of both replicates, at the end of the diafiltration of the intermediate-molecular-mass fraction (GSE_FR30–5_) of the 30 kDa GSE permeate with the 5 kDa MMCO membrane.

Diafiltration GSE_FR30–5_	Diafiltration Intensity (Number of Washings)	Electrical Conductivity (μS/cm)
Replicate 1	7	109
Replicate 2	12	43

**Table 7:** Material balance of the fractionation of the GSE_Pe5_ by the 1 kDa membrane in both replicates.

Pe5	V_Pe5_	TDS_Pe5_	Ms,_Pe5_	ms_,FR5–1_	Pro GSE_cla_	V_Pe1_	TDS_Pe1_	ms_,Pe1_
	(L)	(g/100 mL)	(g)	(g)	(%)	(L)	(g/100 mL)	(g)
Sub-rep 1′	4.0	6.0	240	83.6		2.0	4.3	86
Sub-rep 1″	4.7	6.0	282	98.7		2.4	4.4	106
Rep 1	8.7	6.0	522	182.3	21.1	4.4	4.4	194
Sub-rep 2′	4.0	5.4	216	80.1		2.0	3.9	78
Sub-rep 2″	3.5	5.4	189	58.1		1.8	4.0	72
Rep 2	7.5	5.4	405	138.2	18.0	3.8	3.9	150

V—volume; TDS—total dissolved substances; ms—dry matter; FR—fraction; Pro GSE_clar_—proportion of the clarified GSE; Pe5—5 kDa permeate; Rep—replicate.

**Table 8:** Diafiltration intensity and electrical conductivity achieved in the permeates of the four sub-replicates at the end of the diafiltration of the low-molecular-mass fraction (GSE_FR5–1_) of the 5 kDa GSE permeate with the 1 kDa MMCO membrane.

Diafiltration GSE_FR5–1_	Diafiltration Intensity (Number of Washings)	Electrical Conductivity (μS/cm)
Replicate 1′	12	49.2
Replicate 1″	12	50.1
Replicate 2′	11	48.6
Replicate 2″	11	47.2

**Table 9:** Material balance of the fractionation of the GSE_Pe1_ by solid-phase extraction with FPX66 adsorption resin of replicates 1 and 2 and diafiltration permeates DP_<1_.

	Input Matter			Ouput Matter								
Pe1				Sub-Fraction < 1, w				Sub-Fraction < 1, et				
	V_Pe1_	TDS_Pe1_	ms_Pe1_	V_<1,w_	ms_<1,w_	Rec _FR<1,w_	Pro GSE_cla_	V_<1,et_	ms_<1,et_	Rec _FR<1,et_	Pro GSE_cla_	TRM
	(L)	(g/100 mL)	(g)	(L)	(g)	(%)	(%)	(L)	(g)	(%)	(%)	(%)
Rep1	4.4	4.4	194	6.4	158	81.4	18.3	2.1	12.3	6.4	0.7	87.8
Rep2	3.8	3.9	148	5.8	101	68.2	13.1	2.0	9.1	6.7	0.9	74.0
DP	5.2	3.8	198	7.4	139	70.2	17.0	2.0	11.1	6.0	1.3	76.2
TMB	13.1	4.0	542	20.3	398	73.3	48.4	6.1	32.5	6.2	3.9	79.3

Pe1—1 kDa membrane permeate; V—volume; TDS—total dissolved substances; ms—dry matter; w—water; FR—fraction; Rec—recovery; Pro—proportion of the clarified GSE; Pe5—5 kDa permeate; TRM—total recovered matter; Rep—replicate; DP—diafiltration permeates from the 1 kDa membrane; TMB—total material balance.

## Data Availability

The original contributions presented in this study are included in the article/Supplementary Materials. Further inquiries can be directed to the corresponding author.

## Supplementary Materials

The following supporting information can be downloaded at: https://www.mdpi.com/article/10.3390/membranes15030092/s1, Figure S1: Permeate (blue dots) and concentrate (red dots) TDS kinetics and TDS transfer (orange dots) during ultra/diafiltration of the clarified GSE with the 300 kDa membrane: a) replicate 1 and b) replicate 2; Figure S2: Permeate (blue dots) and concentrate (red dots) electrical conductivity (EC) kinetics and electrolyte transfer (orange dots) during ultra/diafiltration of the clarified GSE with the 300 kDa membrane (replicate 1); Figure S3: Permeate (blue dots) and concentrate (red dots) pH kinetics during ultra/diafiltration of the clarified GSE with the 300 kDa membrane (replicate 2); Figure S4: Hydraulic permeability test of the 300 kDa membrane before (blue line) and after chemical regeneration following the first (red line) and the second (grey line) filtration replicates; Figure S5: Permeate (blue dots) and concentrate (red dots) TDS kinetics and TDS transfer (orange dots) during ultra/diafiltration of the 300 kDa GSE permeate with the 30 kDa membrane: a) replicate 1 and b) replicate 2; Figure S6: Permeate (blue dots) and concentrate (red dots) pH kinetics during ultra/diafiltration of the 300 kDa GSE permeate with the 30 kDa membrane (replicate 2); Figure S7: Hydraulic permeability test of the 30 kDa membrane before (blue line) and after chemical regeneration following the first (red line) and the second (grey line) filtration replicates; Figure S8: Permeability test of the 5 kDa MMCO membrane to the 30 kDa GSE permeate at different pressures; Figure S9: Permeate (blue dots) and concentrate (red dots) TDS kinetics and TDS transfer (orange dots) during ultra/diafiltration of the 30 kDa GSE permeate with the 5 kDa membrane, a) replicate 1 and b) replicate 2; Figure S10: Permeate (blue dots) and concentrate (red dots) pH kinetics during ultra/diafiltration of the 30 kDa GSE permeate with the 5 kDa membrane (replicate 2); Figure S11: Hydraulic permeability test of the 5 kDa membrane before (blue and grey lines) and after chemical regeneration following the first (red line) and the second (green line) filtration replicates; Figure S12: Permeability test of the 1 kDa MMCO membrane to the 5 kDa GSE permeate at different pressures; Figure S13: Permeate (blue dots) and concentrate (red dots) TDS kinetics and TDS transfer (orange dots) during ultra/diafiltration of the 5 kDa GSE permeate with the 1 kDa membrane: a) replicate 1′ and b) replicate 2′; Figure S14: Permeate (blue dots) and concentrate (red dots) pH kinetics during ultra/diafiltration of the 5 kDa GSE permeate with the 1 kDa membrane (replicate 2′); Figure S15: Hydraulic permeability test of the 1 kDa membrane before (blue line) and after chemical regeneration following the 1′ (red line), 1″ (grey line), 2′ (green line) and 2″ (orange line) filtration replicates; Figure S16: Filtration flux kinetic during concentration of the GSE_Pe1_ diafiltration permeates from the 1 kDa membrane treatment by RO; Figure S17: Permeate (blue dots) and concentrate (red dots) TDS kinetics and TDS transfer (orange dots) during concentration of the GSE diafiltration permeates of the 1 kDa membrane treatment (DP_1_) with the RO membrane; Figure S18: Permeate (blue dots) and concentrate (red dots) electrical conductivity kinetics and electrolyte transfer (orange dots) during concentration of the GSE diafiltration permeates of the 1 kDa membrane treatment (DP_1_) with the RO membrane; Figure S19: Permeate (blue dots) and concentrate (red dots) pH kinetics during concentration of the GSE diafiltration permeates of the 1 kDa membrane treatment (DP_1_) with the RO membrane; Figure S20: RP-HPLC chromatogram (280 nm) of 200-fold concentrated RO grape seed extract permeate. GA—gallic acid; C—catechin; EC—epicatechin; Figure S21: Hydraulic permeability test of the 96% NaCl rejection reverse osmosis membrane before (blue line) and after chemical regeneration (red line).
