# Supplementary material for: Fractionation of a Procyanidin-Rich Grape Seed Extract by a Preparative Integrated Ultrafiltration/Reverse Osmosis/Solid-Phase Extraction Procedure
Source: Membranes (Basel). 2025 Mar 14;15(3):92. doi: 10.3390/membranes15030092 (PMC11944240; doi:10.3390/membranes15030092)
Supplement: Supplementary file 1 [file membranes-15-00092-s001.zip › GSE-UF,RO,SPE-SupplData.pdf]

Supplementary data

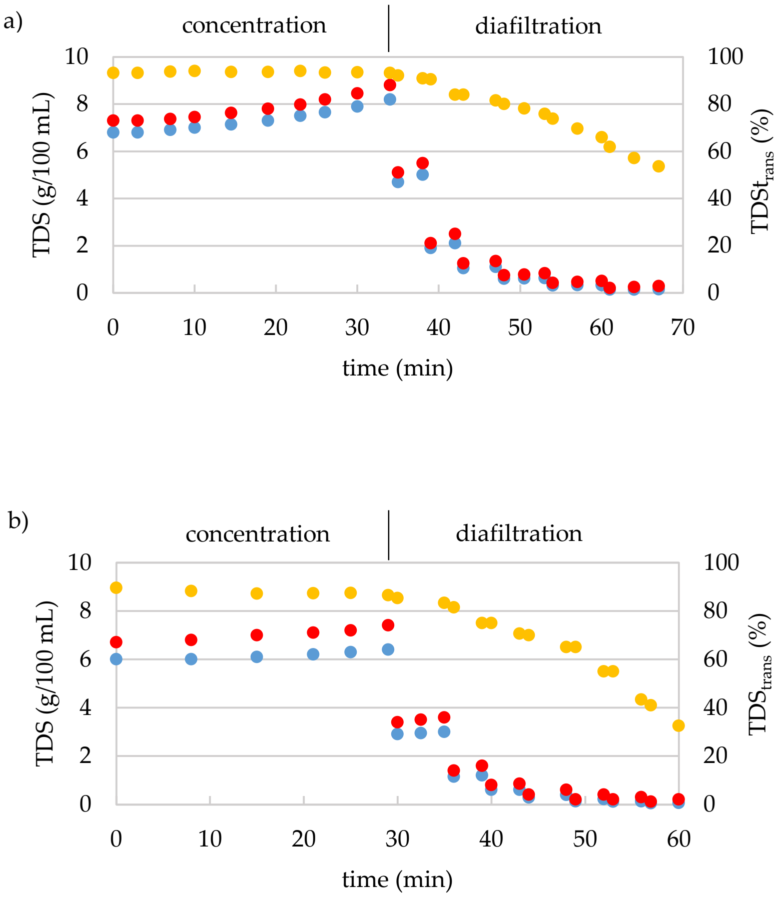

Figure S1. Permeate (blue dots) and concentrate (red dots) TDS kinetics and TDS transfer (orange dots) during ultra/diafiltration of the clarified GSE with the 300 kDa membrane: a) replicate 1 and b) replicate 2.

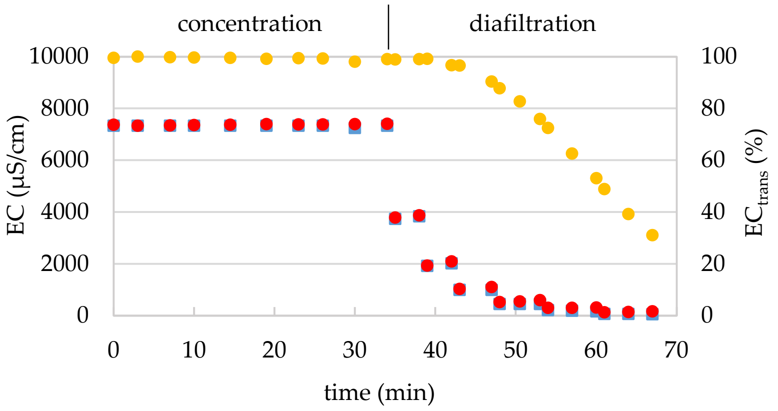

Figure S2. Permeate (blue dots) and concentrate (red dots) electrical conductivity (EC) kinetics and electrolyte transfer (orange dots) during ultra/diafiltration of the clarified GSE with the 300 kDa membrane (replicate 1).

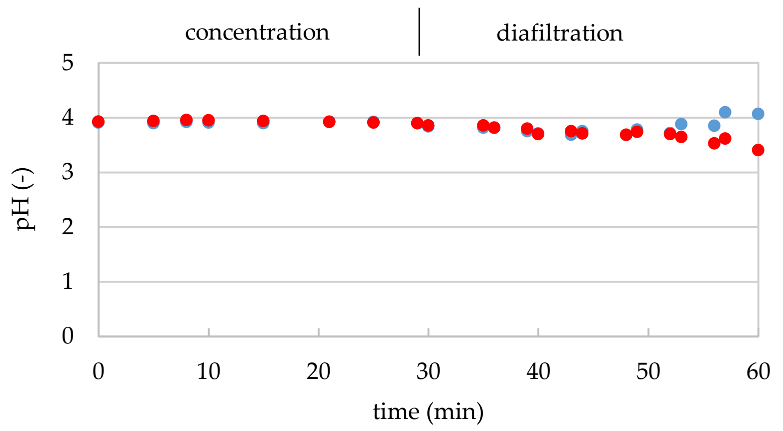

Figure S3. Permeate (blue dots) and concentrate (red dots) pH kinetics during ultra/diafiltration of the clarified GSE with the 300 kDa membrane (replicate 2).

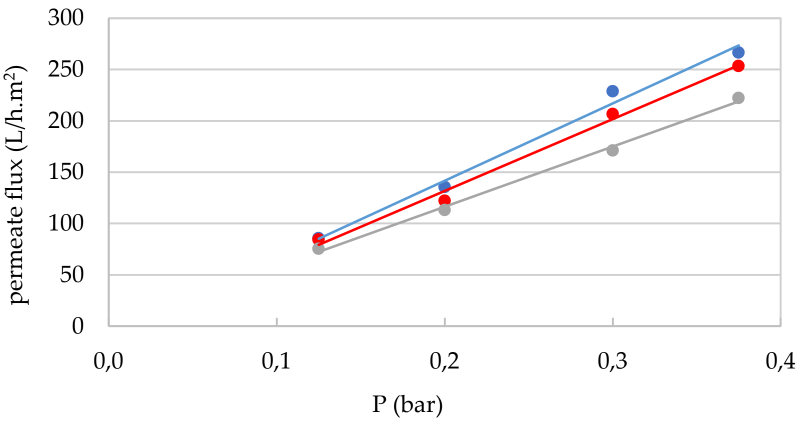

Figure S4. Hydraulic permeability test of the 300 kDa membrane before (blue line) and after chemical regeneration following the first (red line) and the second (grey line) filtration replicates.

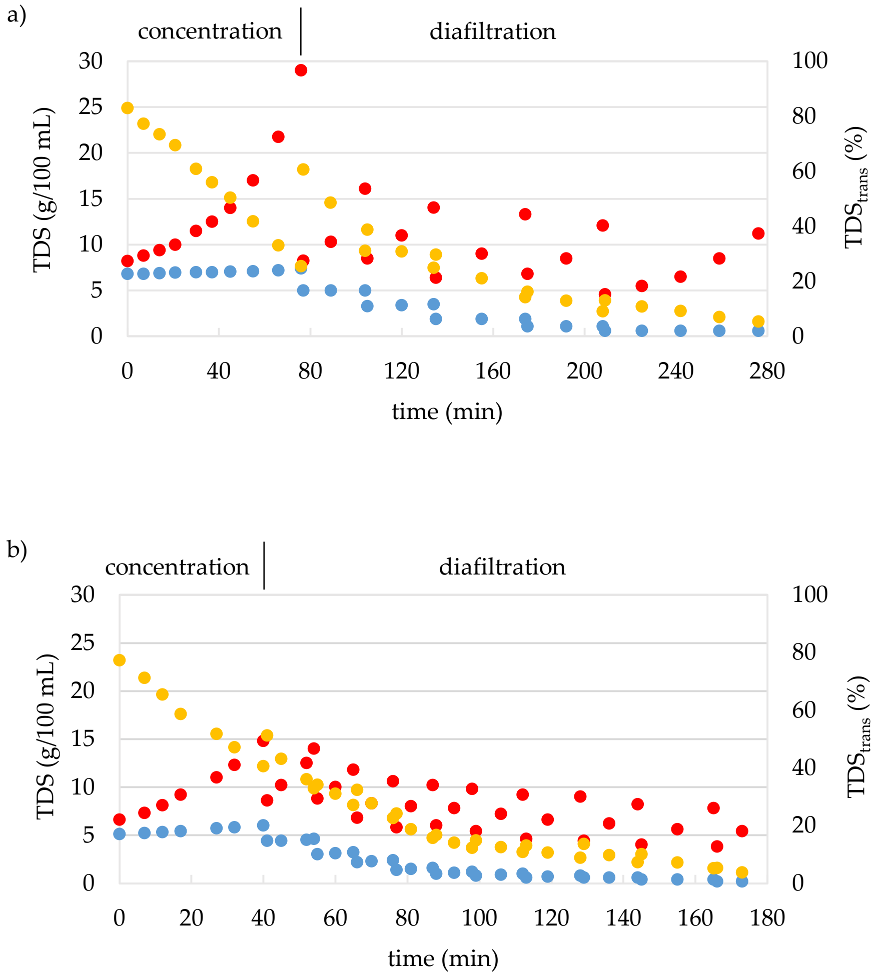

Figure S5. Permeate (blue dots) and concentrate (red dots) TDS kinetics and TDS transfer (orange dots) during ultra/diafiltration of the 300 kDa GSE permeate with the 30 kDa membrane: a) replicate 1 and b) replicate 2.

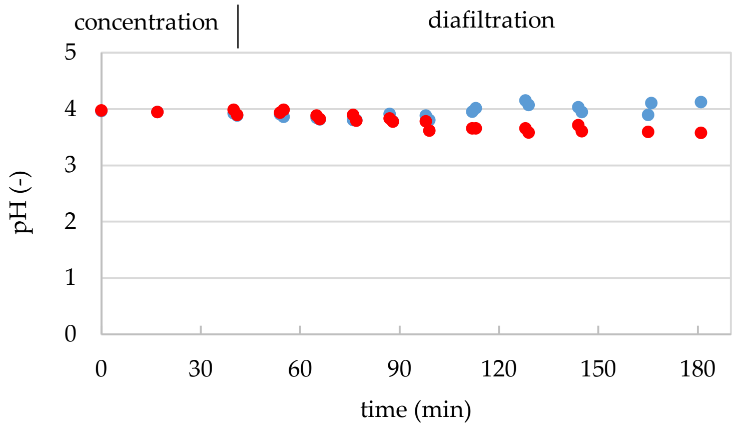

Figure S6. Permeate (blue dots) and concentrate (red dots) pH kinetics during ultra/diafiltration of the 300 kDa GSE permeate with the 30 kDa membrane (replicate 2).

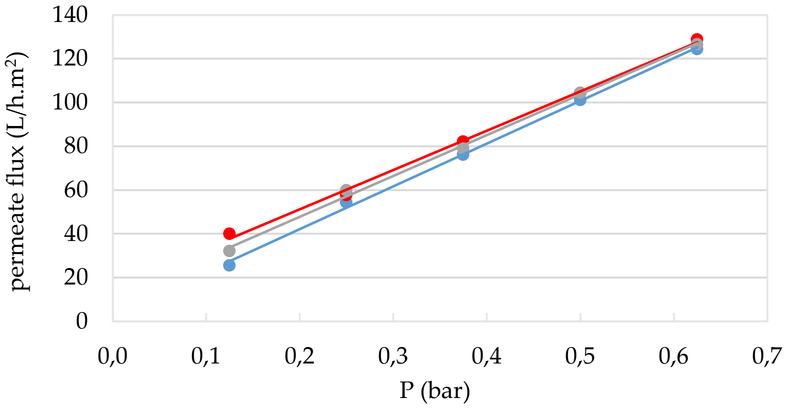

Figure S7. Hydraulic permeability test of the 30 kDa membrane before (blue line) and after chemical regeneration following the first (red line) and the second (grey line) filtration replicates.

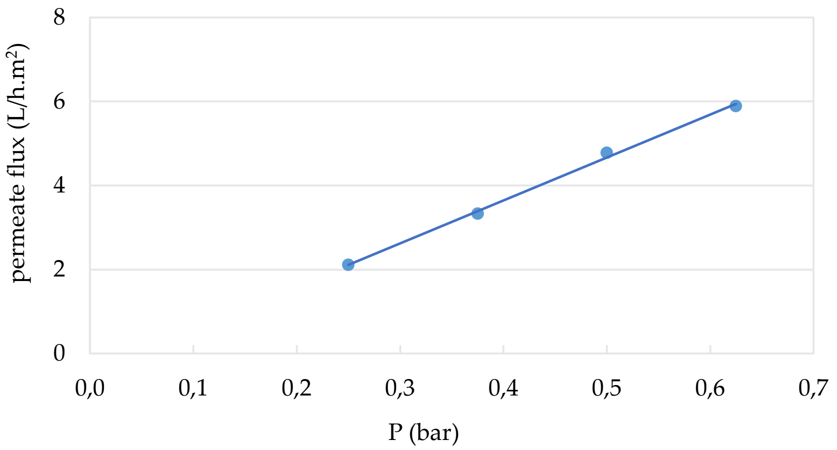

Figure S8. Permeability test of the 5 kDa MMCO membrane to the 30 kDa GSE permeate at different pressures.

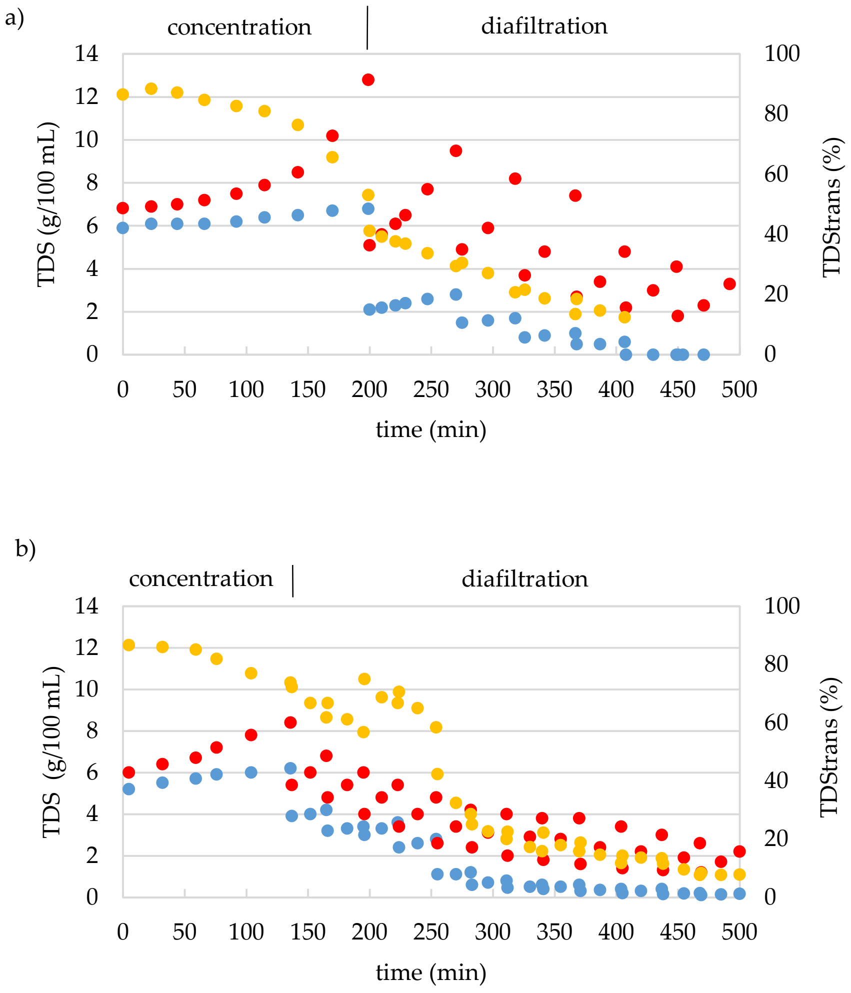

Figure S9. Permeate (blue dots) and concentrate (red dots) TDS kinetics and TDS transfer (orange dots) during ultra/diafiltration of the 30 kDa GSE permeate with the 5 kDa membrane, a) replicate 1 and b) replicate 2.

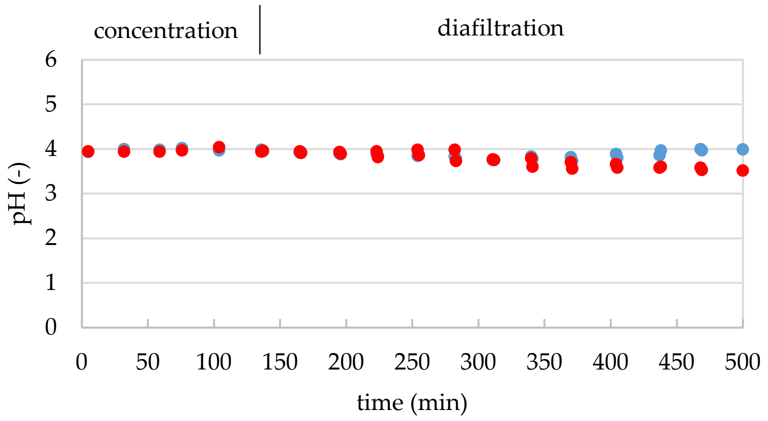

Figure S10. Permeate (blue dots) and concentrate (red dots) pH kinetics during ultra/diafiltration of the 30 kDa GSE permeate with the 5 kDa membrane (replicate 2).

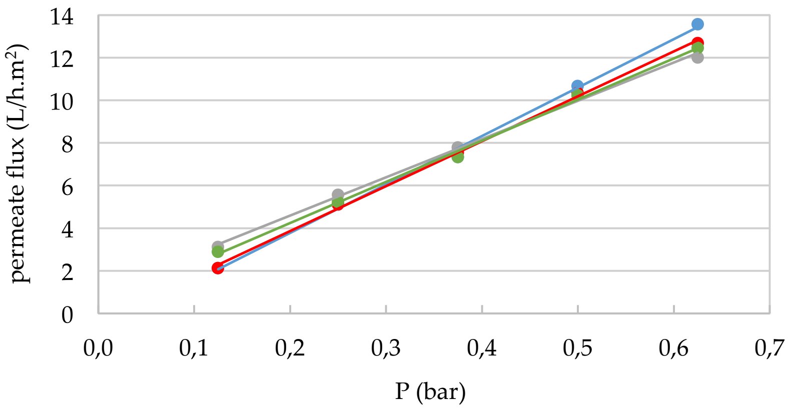

Figure S11. Hydraulic permeability test of the 5 kDa membrane before (blue and grey lines) and after chemical regeneration following the first (red line) and the second (green line) filtration replicates.

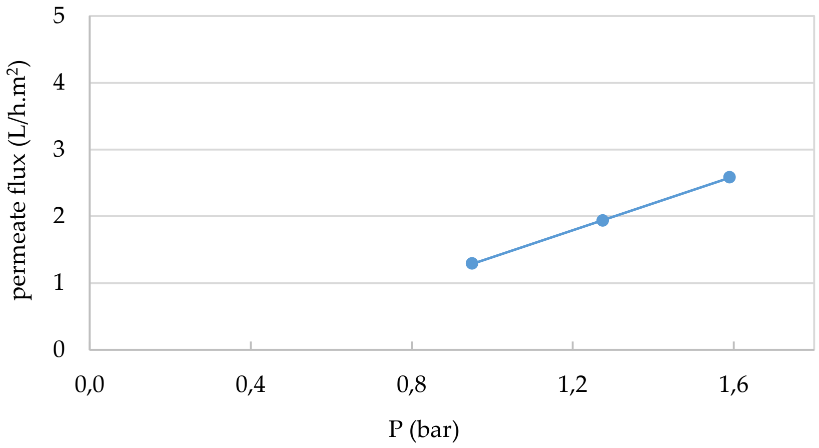

Figure S12. Permeability test of the 1 kDa MMCO membrane to the 5 kDa GSE permeate at different pressures.

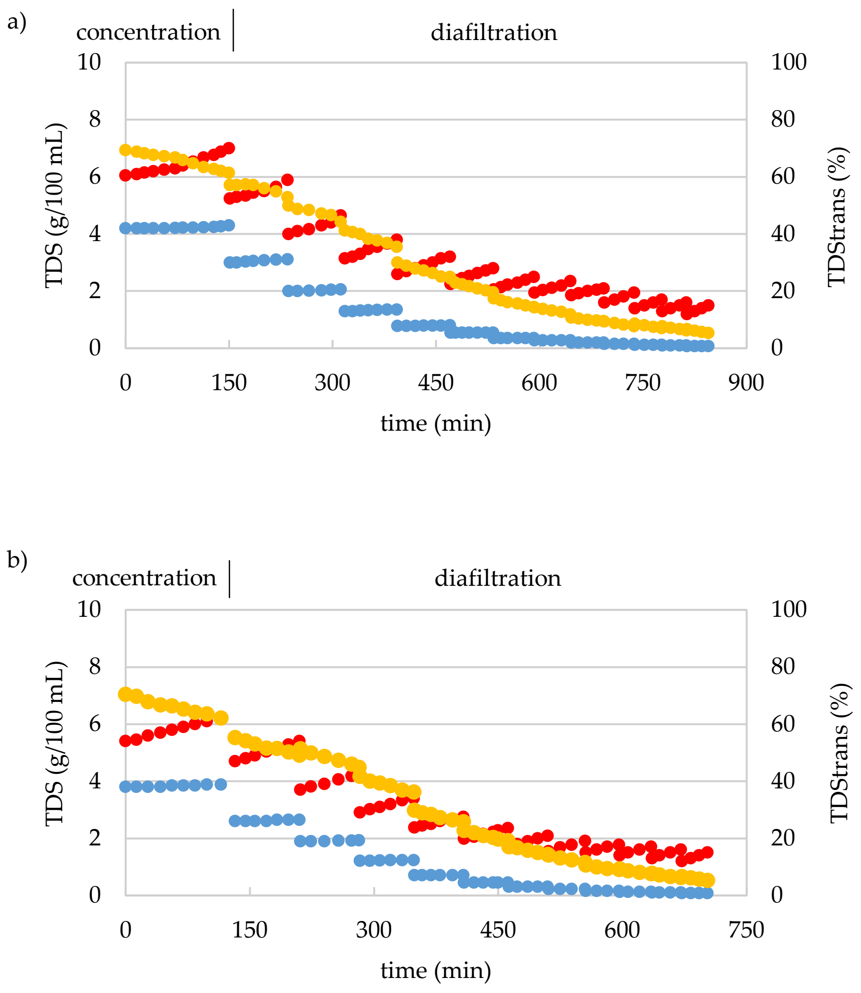

Figure S13. Permeate (blue dots) and concentrate (red dots) TDS kinetics and TDS transfer (orange dots) during ultra/diafiltration of the 5 kDa GSE permeate with the 1 kDa membrane: a) replicate 1′ and b) replicate 2′.

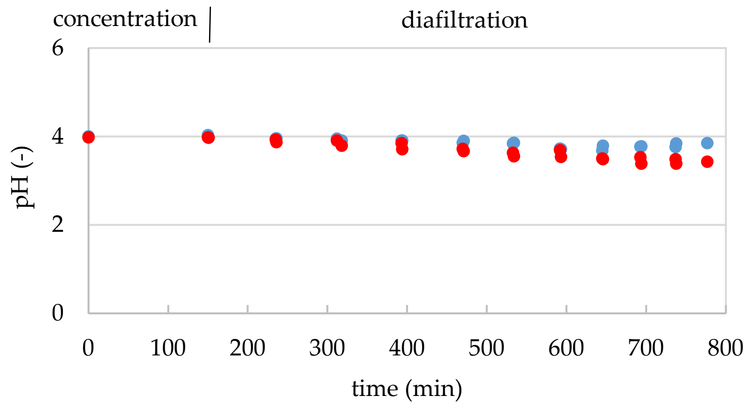

Figure S14. Permeate (blue dots) and concentrate (red dots) pH kinetics during ultra/diafiltration of the 5 kDa GSE permeate with the 1 kDa membrane (replicate 2′).

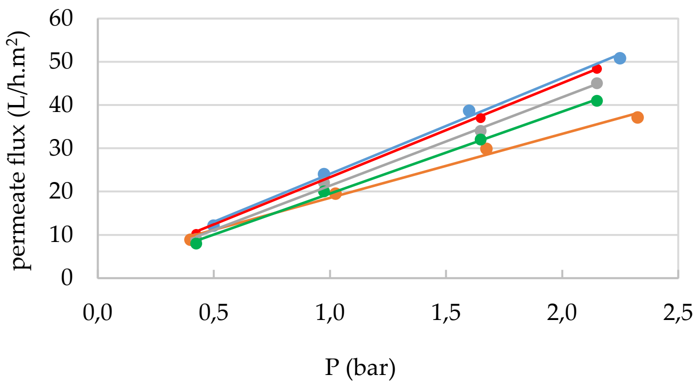

Figure S15. Hydraulic permeability test of the 1 kDa membrane before (blue line) and after chemical regeneration following the 1′ (red line), 1′′ (grey line), 2′ (green line) and 2′′ (orange line) filtration replicates.

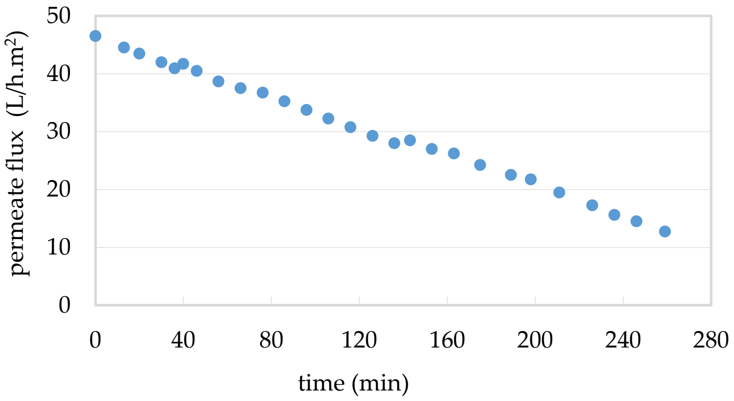

Figure S16. Filtration flux kinetic during concentration of the GSE_{Pe1} diafiltration permeates from the 1 kDa membrane treatment by RO.

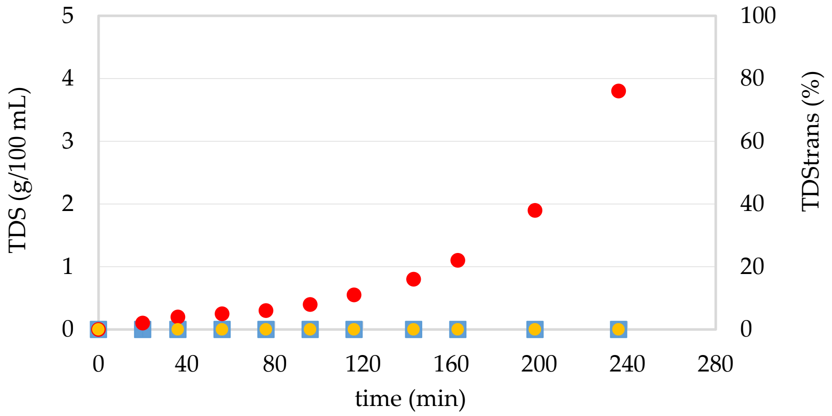

Figure S17. Permeate (blue dots) and concentrate (red dots) TDS kinetics and TDS transfer (orange dots) during concentration of the GSE diafiltration permeates of the 1 kDa membrane treatment (DP_1) with the RO membrane.

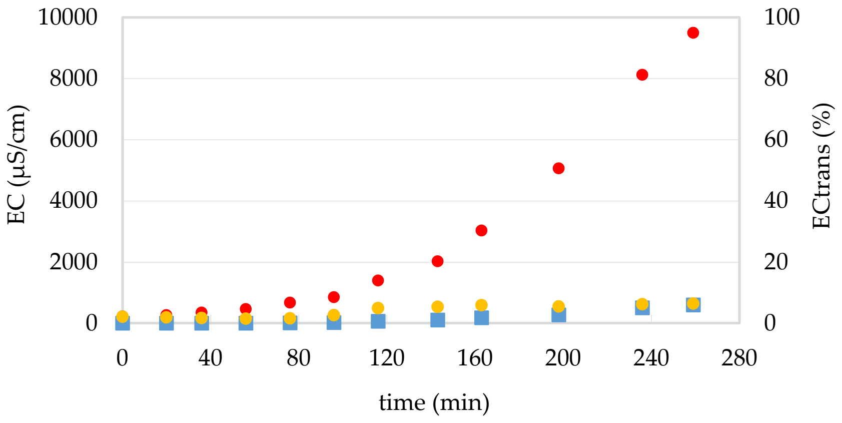

Figure S18. Permeate (blue dots) and concentrate (red dots) electrical conductivity kinetics and electrolyte transfer (orange dots) during concentration of the GSE diafiltration permeates of the 1 kDa membrane treatment (DP_1) with the RO membrane.

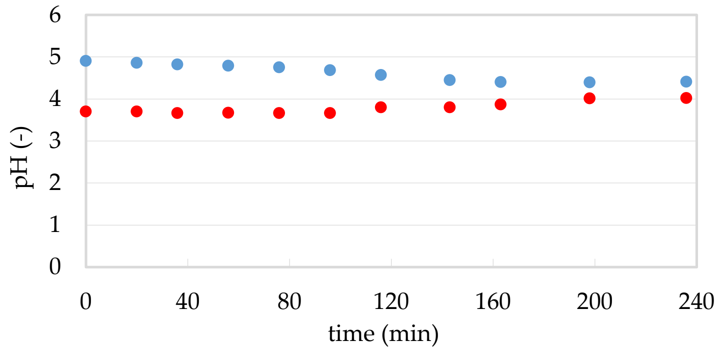

Figure S19. Permeate (blue dots) and concentrate (red dots) pH kinetics during concentration of the GSE diafiltration permeates of the 1 kDa membrane treatment (DP_1) with the RO membrane.

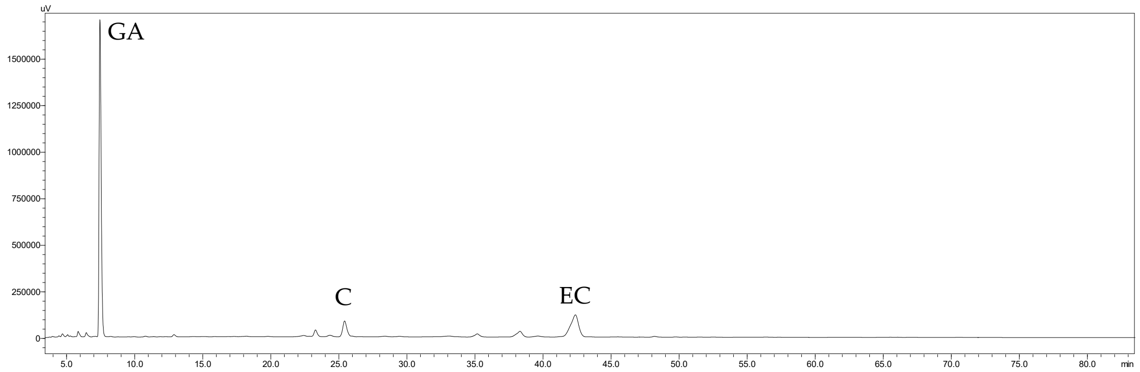

Figure S20. RP-HPLC chromatogram (280 nm) of 200-fold concentrated RO grape seed extract permeate, GA – gallic acid, C – catechin, EC - epicatechin.

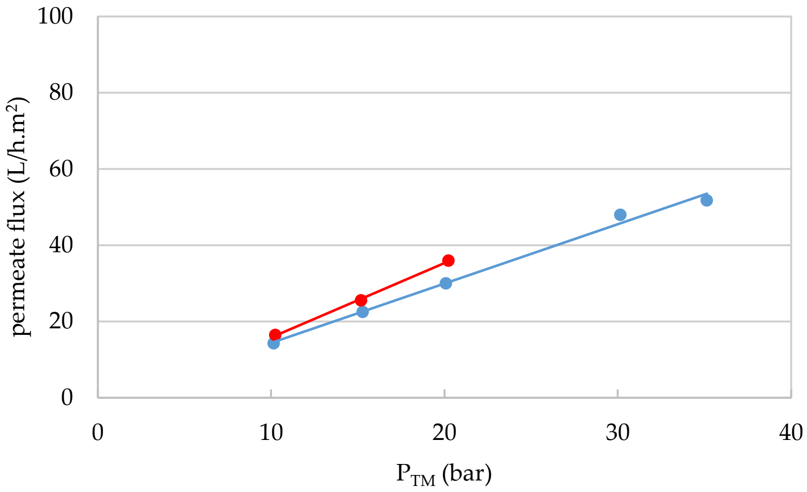

Figure S21. Hydraulic permeability test of the 96% NaCl rejection reverse osmosis membrane before (blue line) and after chemical regeneration (red line).
